# The secreted protein SPARCL1 suppresses tumor progression in papillary thyroid carcinoma via SLC3A2-mediated ferroptosis

**DOI:** 10.1530/ERC-26-0018

**Published:** 2026-06-08

**Authors:** Fengping Wu, Jinkang Zhang, Xiaoli Zhang, Dongkun Xu, Shuanghua Cheng, Qian Liu, Yuxin Gan, Lifang Ren, Haocheng Yang, Kun Zhang, Xuliang Xia, Zhiqiang Jiang, Yuhong Shi, Wenling Tu

**Affiliations:** ^1^ The Second Affiliated Hospital of Chengdu Medical College, China National Nuclear Corporation 416 Hospital, Chengdu, China; ^2^ School of Bioscience and Technology, Chengdu Medical College, Chengdu, China; ^3^ The Seventh People’s Hospital, Affiliated Cancer Hospital of Chengdu Medical College, Chengdu, China

**Keywords:** SPARCL1, papillary thyroid carcinoma, invasion and metastasis, therapeutic candidate, ferroptosis

## Abstract

Papillary thyroid carcinoma (PTC) is the most prevalent type of thyroid malignancy, where invasive growth and distant metastasis contribute to a worsened patient prognosis. SPARCL1, a known tumor suppressor gene in various cancers, encodes a secreted glycoprotein. Preliminary research suggests that elevated expression of SPARCL1 is correlated with a better prognosis in PTC, highlighting its potential as an innovative restorative therapeutic agent, but its specific role and underlying mechanisms remain unclear. This study aimed to investigate the impact of SPARCL1 on the invasion and metastasis of PTC cells by constructing an overexpression model of SPARCL1 in PTC cells through viral transduction, collecting the supernatant proteins from the PTC cell model and adding them to PTC cells, and introducing recombinant SPARCL1 protein into PTC cells. In addition, we conducted animal experiments to detect the effects of SPARCL1 on the subcutaneous tumor formation and multi-organ metastasis of PTC cells in mice, as well as the inhibitory effect of recombinant SPARCL1 on tumors formed by PTC cells. The findings revealed that overexpression of the SPARCL1 gene, the secretory form of the SPARCL1 protein, and the recombinant SPARCL1 protein all significantly inhibited the malignant biological behaviors of PTC cells. Overexpression of SPARCL1 can inhibit the subcutaneous growth of tumor cells and their metastasis to the lungs, liver, and kidneys of mice, and recombinant SPARCL1 protein can also inhibit the growth of subcutaneous tumors. Mechanistically, SPARCL1 appears to mediate its anti-tumor effects through the SLC3A2-mediated ferroptosis pathway, suggesting a novel mechanism by which SPARCL1 influences PTC progression. In summary, SPARCL1 inhibits the proliferation, invasion, and metastasis of PTC, highlighting its potential as a candidate for innovative restorative therapies in PTC treatment.

## Introduction

Papillary thyroid carcinoma (PTC) stands as the most prevalent malignancy within the endocrine system, constituting 70–80% of all thyroid cancers recorded (1, 2). The clinical evolution of PTC generally starts with an indolent localized growth but can dynamically progress – via extrathyroidal invasion and lymphatic spread – into aggressive lateral neck metastasis and subsequent systemic recurrence in specific populations (1, 3). Although the overall prognosis for differentiated thyroid cancer is highly favorable, individuals aged 55 and older with lateral cervical lymph node metastasis (N1b) experience a significantly elevated risk of both relapse and mortality (4, 5). Invasive growth and the ability to metastasize distantly are defining traits of PTC’s aggressive biology and are pivotal factors in the unfavorable outcomes observed in affected individuals. Therefore, understanding the underlying mechanisms of PTC’s invasion and metastasis and finding therapeutic approaches at the molecular level are urgent tasks in the fight against this disease.

Oncogenes and tumor-suppressor genes play a very important role in the occurrence and development of tumors (6, 7, 8). Abnormal expression of these genes may affect the biological functions of tumor cells, such as proliferation, apoptosis, migration, and invasion (9, 10). Recent studies have shown that tumor suppressor genes may play a more important role in anti-tumor responses than oncogenes (6, 7). Several studies have shown that many tumor suppressor genes have abnormal changes in PTC, such as significant mutations in TP53 (11), CHEK2 (12), and RASAL1 (13), and high methylation of the promoter region of PTEN (14). These changes lead to inactivation or low expression of these tumor suppressor genes, indirectly promoting the occurrence of tumors. Conventional treatments targeting oncogenes typically rely on targeted inhibition or gene knockdown strategies, which may inadvertently deprive cells of the gene’s baseline physiological functions. Therefore, an innovative restorative strategy – focusing on reinstating tumor suppressor function – may represent a safer alternative. It is reported that more than half of cancers are driven by mutations in tumor suppressor genes; however, almost all of the approximately 80 anti-tumor targeted drugs currently used in clinical practice target proteins encoded by oncogenes. Consequently, only 2–13% of cancer patients are eligible to receive these targeted treatments (15, 16). In 2023, Professor Lu discovered that arsenic trioxide (ATO) can restore the function of the tumor suppressor p53 and reduce residual microlesions in leukemia patients (17). This landmark study achieved the first functional restoration of a tumor suppressor protein in the human body, providing a compelling paradigm for future innovative restorative therapies in oncology. Similarly, in the research on PTC treatment, exploiting tumor suppressor genes as additional or innovative therapeutic agents has also shown highly promising results (18, 19). Overexpression of lncAB, an inhibitory long noncoding RNA, can inhibit the proliferation of PTC cells *in vitro* and *in vivo* (20). In addition, another study showed that overexpression of FTO, a tumor suppressor gene, could inhibit the proliferation, migration, and invasion of PTC cells *in vitro* and *in vivo* (21). In summary, these studies demonstrate the great potential of tumor suppressor genes as candidates for innovative restorative therapies in the treatment of PTC.

SPARCL1 is an acidic, cysteine-rich secreted extracellular glycoprotein belonging to the SPARC family, which not only acts as a key regulator of glucose and extracellular matrix metabolism (22, 23, 24) but also plays a vital role in regulating cell adhesion, migration, and proliferation (25, 26). The gene encoding SPARCL1 is localized to human chromosome 4q22–25 (27), a region that is frequently deleted in lung cancer (28, 29), breast cancer (30), bladder cancer (31), thyroid cancer (32), and other cancers (27). Research indicates that SPARCL1 significantly influences tumorigenesis. In numerous cancer types, diminished levels of SPARCL1 have been observed, potentially correlating with heightened tumor invasiveness and metastatic capability. For instance, in breast and lung cancers, SPARCL1 expression levels are intimately tied to the tumor’s clinical stage, lymphatic spread, and patient survival outcomes (33). In cervical cancer, SPARCL1 can downregulate the expression of osteopontin, thereby inhibiting cell proliferation, migration, and invasion (34). In prostate cancer, SPARCL1 inhibits the metastasis and invasion of cancer cells by binding to collagen in the extracellular matrix and the cytoskeleton within the cell (35). However, to our knowledge, the specific role and mechanism of SPARCL1 have not been reported in PTC.

The expression pattern of SPARCL1 in PTC and its impact on the progression of the disease have not yet been extensively studied and warrant further exploration. In our preliminary research, analysis of The Cancer Genome Atlas (TCGA) database for PTC RNA-seq data and clinical information confirmed that SPARCL1 is abnormally lowly expressed in PTC and that patients with lower SPARCL1 expression have shorter disease-free survival. Further validation at the protein level confirmed that SPARCL1 is less expressed in PTC than in normal thyroid tissue. In the present study, our *in vitro* experiments demonstrated that overexpressing SPARCL1 in PTC cells, or treating them with either SPARCL1-containing supernatant or recombinant SPARCL1 protein, significantly inhibited the oncogenic behaviors of PTC cells. Furthermore, our animal models revealed that SPARCL1 suppresses the proliferation of PTC cells and their distant metastasis to organs such as the lungs, liver, and kidneys *in vivo*. These findings suggest that the downregulation of SPARCL1 is involved in the malignant biological behavior of PTC. Most importantly, overexpression of SPARCL1 or supplementation of SPARCL1 protein represents a new and promising approach to treat PTC. Mechanistic experiments showed that SPARCL1 inhibited the malignant biological behavior of PTC cells, which may be achieved through the SLC3A2-mediated ferroptosis pathway. Therefore, this study aims to elucidate the role and underlying mechanisms of SPARCL1 in PTC invasion and metastasis, highlighting its potential as an innovative restorative therapeutic strategy for PTC.

## Materials and methods

### Collection of thyroid tumor tissue and adjacent normal tissue from PTC patients

After obtaining approval from the Ethics Committee of the Second Affiliated Hospital of Chengdu Medical College, China National Nuclear Corporation 416 Hospital, and informed consent from the patients, we collected thyroid tumor tissues and adjacent normal tissues from 12 PTC patients at the Second Affiliated Hospital of Chengdu Medical College, China National Nuclear Corporation 416 Hospital. Then we cut the tissues into small pieces, added protein lysis buffer to them, and put them into the tissue grinder for thorough grinding and extraction of total protein for subsequent experiments.

### Construction of PTC cell models overexpressing SPARCL1

First, 3 × 10^5^ TPC-1 cells and 5 × 10^5^ BCPAP cells were inoculated into six-well plates. After 24 h, the culture medium was replaced with serum-free culture medium mixed with HBLV-SPARCL1-3xflag-PURO lentivirus or HBLV-PURO lentivirus (Hanbio, China), and an appropriate amount of Polybrene (Hanbio, China) was added. After 24 h of lentiviral infection, the medium in the six-well plate was replaced with new complete medium, and appropriate puromycin (Hanbio, China) was added. After 48 h of puromycin addition, proteins from some cells were extracted, and a western blot analysis was performed to determine whether the PTC cell models overexpressing SPARCL1 were successfully constructed.

### Real-time quantitative PCR (RT-qPCR)

Cells are lysed with TRIzol (MRC, USA), and RNA is extracted with chloroform, isopropanol, and ethanol before being dissolved in water without enzymes. RNA, primers, RNase inhibitors, reverse transcriptase, dNTP mixture, and other reverse transcription reagents (TIANGEN, China) are added to the PCR tube and placed in the PCR instrument for incubation to produce cDNA. In 8-tube strips, primers, mix, cDNA, and ddH_2_O are added, and real-time PCR is performed to detect expression levels using SuperReal PreMix Plus (TIANGEN, China). The relative expression levels of genes were calculated by 2^−^^Δ^^Δ^^Ct^. Primer sequences are provided in Supplementary Material 1 (see section on Supplementary materials given at the end of the article).

### mRNA-seq and bioinformatics analysis

For PTC mRNA-seq data, we first downloaded the mRNA expression data of PTC from the TCGA database (https://portal.gdc.cancer.gov/). The differentially expressed genes analysis was performed by the ‘wilcox.test’ function in the R package ‘stats’, and genes that satisfied |log_2_FC|≥1.5 and adjusted *P* value < 0.05 were identified as DEGs. The results of the differentially expressed gene analysis were visualized as a volcano plot using the R package ‘ggplot2’. Then, the gene ontology (GO) enrichment analysis of DEGs was performed using the R package ‘clusterProfiler’, and the results were presented using the R package ‘ggraph’.

We used TRIzol (MRC, USA) to extract RNA from BCPAP-NC and BCPAP-SPARCL1 cells and then performed mRNA sequencing to obtain sequencing data. The detailed steps of mRNA sequencing can be found in our previous study (36). The raw data of sequencing have been uploaded to the SRA database (https://www.ncbi.nlm.nih.gov/sra), and the SRA accession number was PRJNA1122957. The R package ‘limma’ was used to perform differentially expressed gene analysis on mRNA sequencing data, and genes satisfying |log_2_FC|≥1 and adjusted *P* value < 0.05 were identified as differentially expressed genes (DEGs). The results of differentially expressed gene analysis were visualized as volcano plots using the R package ‘ggplot2’. Then, we performed GO enrichment analysis and Kyoto Encyclopedia of Genes and Genomes (KEGG) enrichment analysis on DEGs using the R package ‘clusterProfiler’. In addition, Gene Set Variation Analysis (GSVA) was performed to calculate enrichment scores for each sample in the mRNA-seq data based on the gene set of 18 cell death modes (Supplementary Material 1) using the R package ‘GSVA’ and then visualized using the R package ‘pheatmap’.

### Western blot

First, the concentration of the extracted protein was quantified using the BCA protein assay kit (CWBIO, China). Then, an appropriate amount of protein was mixed with the loading buffer and heated at 95°C for 10 min. After loading, the voltage of electrophoresis was set to 120 V for about 40 min. The current of the transfer was set to 300 mA for 2 h. After the transfer, the PVDF membrane was blocked with 5% skim milk for 2 h at room temperature. After washing the PVDF membrane three times with TBST, it was incubated with the primary antibody at 4°C overnight. After washing the PVDF membrane again, the membrane was treated with the secondary antibody at room temperature for 2 h. Finally, TBST was used to wash off the excess secondary antibody on the PVDF, and the results were visualized and recorded using the chemiluminescent gel imaging system. Primary antibodies against SPARCL1 (Proteintech, USA, Cat#: 13517-1-AP), SLC3A2 (Proteintech, USA, Cat#: 15193-1-AP), FACL4 (Abcam, UK, Cat#: ab155282), GPX4 (Affinity Biosciences, USA, Cat#: DF6701), Flag tag (Zenbio, China, Cat#: 390002), and β-actin (Affinity Biosciences, USA, Cat#: T0022) were used. Secondary antibodies against rabbit IgG (Affinity Biosciences, USA, Cat#: #S0001) and mouse IgG (Affinity Biosciences, USA, Cat#: #S0002) were used in this experiment.

### CCK-8 assay

Cells were plated in a 96-well plate with 3 × 10^3^ TPC-1 cells per well and 5 × 10^3^ BCPAP cells per well. After the cells had adhered, CCK-8 reagent (APExBIO, USA) was added at 0, 24, 48, and 72 h, followed by incubation for 2 h. Optical density values were then measured at 450 nm using a microplate reader.

### Colony formation assay

For the clonogenic assay, cells were plated in a six-well plate at a density of 500 cells per well. Growth was observed daily, and once the colonies had fully formed, they were stained with crystal violet and photographed. The number of clones was obtained by counting with the ImageJ software.

### Transwell migration assay

First, the cells were starved in serum-free medium for 24 h. Then, 600 μL serum-containing medium was added to the lower chamber and serum-free medium-suspended cells were added to the upper chamber. The number of cells per well was 5 × 10^3^, and 100 μL of cell suspension were added to each well. Staining and observation were performed after 3 days of culture for TPC-1 and 2 days for BCPAP. The stained cells that migrated to the bottom of the well were counted using ImageJ software.

### Transwell invasion assay

Cells were serum-starved for 24 h in serum-free medium. The Matrigel (Corning, USA) was diluted at a ratio of 1:8, and 100 μL of the diluted Matrigel were added to each well. The gel was allowed to polymerize at 37°C for 3 h. Then, 600 μL of serum-containing medium were added to the lower chamber, and 100 μL of serum-free medium-suspended cells at a density of 3 × 10^4^ cells per well were added to the upper chamber. For TPC-1 cells, staining and observation were performed after 3 days of culture, whereas for BCPAP cells, staining and observation occurred after 2 days of culture. The stained cells that invaded the bottom of the well were counted using ImageJ software.

### Cell apoptosis

First, cells were inoculated in six-well plates, with a TPC-1 inoculation density of 3 × 10^5^ cells/well and a BCPAP inoculation density of 5 × 10^5^ cells/well. After 24 h, the cells were detached from the six-well plates, centrifuged, and washed twice with phosphate-buffered saline (PBS). Then, 5 μL of Annexin V and PI staining solution (YEASEN, China) were added and incubated at room temperature in the dark for 10 min. Finally, the apoptotic rate was detected and analyzed by flow cytometry. Apoptotic cells are cells that are positive for Annexin V staining.

### Preparation and measurement of cell supernatant

After culturing PTC cells infected with SPARCL1 and control lentivirus for 2 days, serum-free medium (containing 1% penicillin-streptomycin) was replaced and the cells were cultured for another 2 days. The supernatant from each group of cells was collected and subjected to preliminary centrifugation to remove cell precipitates and debris. The supernatant was then concentrated tenfold using Ultra-15 centrifugal filter units (Thermo Fisher Scientific, USA) and stored. Dilute the concentrated supernatant to an appropriate concentration for cell culture. Determine the protein concentration of the concentrated supernatant using the BCA assay kit. Adjust the concentration of the control group and overexpression group supernatants to the same level and conduct the experiment.

### Preparation of recombinant SPARCL1 protein stock solution

The recombinant SPARCL1 dry powder (FineTest, China) was dissolved in sterile water and aliquoted to prevent damage to the protein due to repeated freeze–thaw cycles. The aliquots were thawed prior to use in experiments. For TPC-1 cell experiments, the protein concentration used was 2 μg/mL, while for BCPAP cell experiments, the protein concentration used was 4 μg/mL.

### Immunoprecipitation/liquid chromatography mass spectrometry (Co-IP/MS)

To identify interacting proteins of SPARCL1, we performed Co-IP/LC-MS and analyzed proteins precipitated by Flag tag antibodies (Zenbio, China, Cat#: 390002) in BCPAP-NC and BCPAP-SPARCL1 cells. In brief, according to the above immunoprecipitation steps, magnetic beads were used to enrich the SPARCL1 protein and its interacting proteins to obtain Co-IP products. Dithiothreitol was added to the Co-IP product for reduction for 30 min; iodoacetamide was added, and the mixture was incubated at room temperature in the dark for 15 min, and then trypsin was added for enzymatic digestion overnight. The EASY-nLC 1200 ultrahigh-performance liquid chromatography system (Thermo Fisher Scientific, USA) was used to separate the cleaved peptides. In the liquid chromatography system, mobile phase A is an aqueous solution containing 0.1% formic acid and 2% acetonitrile; mobile phase B is an aqueous solution containing 0.1% formic acid and 90% acetonitrile. The peptide fragments were isolated by the UHPLC system and then introduced into the NSI ion source for ionization, subsequently entering the Orbitrap Exploris 480 mass spectrometer (Thermo Fisher Scientific, USA) for analysis.

### Co-immunoprecipitation

The protein of the cells was extracted with universal protein lysis buffer (BioTeke, China) and quantified by the BCA method (CWBIO, China). The solution containing 1,000 μg of protein was prepared, to which 5 μg antibody against the Flag tag (Zenbio, China, Cat#: 390002), SPARCL1 (Proteintech, USA, Cat#: 13517-1-AP), or SLC3A2 (Proteintech, USA, Cat#: 15193-1-AP) was added. The mixture was then incubated at 4°C with gentle rotation for 12 h. After 12 h, 30 μL of magnetic beads (MedChemExpress, USA) were added to the above mixture and incubated with rotation at 4°C for 6 h. Then, the proteins that were not bound to the magnetic beads were eluted, and the remaining proteins bound to the magnetic beads were added to the loading buffer, boiled, and subjected to SDS-PAGE. Finally, the electrophoresed protein were transferred to a PVDF membrane, blocked for 2 h, incubated with the corresponding primary and secondary antibodies, and chemiluminescence detection was performed. Primary antibodies against SPARCL1 (Proteintech, USA, Cat#: 13517-1-AP), SLC3A2 (Proteintech, USA, Cat#: 15193-1-AP), Flag tag (Zenbio, China, Cat#: 390002), and β-actin (Affinity Biosciences, USA, Cat#: T0022) were used in this experiment. Secondary antibodies against rabbit IgG (Affinity Biosciences, USA, Cat#: #S0001) and mouse IgG (Affinity Biosciences, USA, Cat#: #S0002) were used in this experiment.

### Chemical treatments

To evaluate whether SPARCL1 plays a role in PTC cells through the ferroptosis pathway, we plated 2000 BCPAP cells and 3000 TPC-1 cells into 96-well plates and then treated them with 0.5 μM RSL3 (MCE, China), 10 μM Fer-1 (MCE, China), and a certain amount of recombinant SPARCL1 protein on the next day. After 48 h, the cell activity of these cells was detected by the CCK-8 assay. BCPAP cells and TPC-1 cells were treated with 4 and 2 μg/mL recombinant SPARCL1 protein, respectively. In addition, we also conducted related experiments on PTC cells overexpressing SPARCL1. The steps are as follows: 2000 BCPAP-NC or BCPAP-SPARCL1 cells and 3000 TPC-1-NC cells or TPC-1-SPARCL1 cells were plated into 96-well plates, and then treated with 0.5 μM RSL3 and 10 μM Fer-1 on the next day. After 48 h, the cell activity was detected by the CCK-8 assay.

### Hematoxylin and eosin staining

First, the mouse tumor tissue was cut into small pieces and fixed in 4% paraformaldehyde for 24 h. Then, different concentrations of ethanol and xylene were used to dehydrate and make the tumor tissue transparent. The tissue was then embedded in paraffin to form a wax block, and the embedded wax block was cut into 5 μm slices using a microtome. The sections were dewaxed with xylene and different concentrations of ethanol. Then, the sections were immersed in hematoxylin for 5 min and rinsed with water to remove excess dye. Subsequently, the sections were immersed in eosin for 3 min and rinsed with water to remove excess dye. The sections were immersed in different concentrations of ethanol solutions, cleared with xylene, and dried. Finally, neutral resin was added to the sections and covered with a glass slide, and the sections were placed under a microscope for observation and analysis.

### Immunohistochemical staining

First, the sections mentioned above were immersed in xylene and different concentrations of ethanol for dewaxing and hydration, then heated in a microwave oven for antigen retrieval, and then blocked with hydrogen peroxide for 10 min. Subsequently, the sections were washed three times with PBS, incubated with 5% BSA for 10 min, and then the liquid was discarded and the primary antibody was added at 4°C overnight. The next day, the sections were washed three times with PBS and incubated with the secondary antibody at room temperature for one hour. The sections were then washed with PBS, and diaminobenzidine was added for color reaction. After the color reaction was completed, the sections were washed three times with PBS and counterstained with hematoxylin. Finally, the sections were mounted with the neutral resin and then observed and analyzed under a microscope. Primary antibodies against SPARCL1 (Proteintech, USA, Cat#: 13517-1-AP) and Ki67 (Servicebio, China, Cat#: GB121141) were used in this experiment. Secondary antibodies against rabbit IgG (Servicebio, China, Cat#: GB23204) were used in this experiment.

### Animal experiments

All mouse studies were conducted under the approval of the Animal Ethics Committee of the Second Affiliated Hospital of Chengdu Medical College, China National Nuclear Corporation 416 Hospital. Male NCG nude mice aged four to six weeks were procured from Cyagen Biosciences Inc (China). For subcutaneous tumor formation experiments, first prepare 50 μL of Matrigel and 50 μL of 1 × 10^7^ cells for each mouse, and then mix the cells and Matrigel thoroughly. These cells were injected subcutaneously into NCG mice, and the volume of these tumors was measured using a vernier caliper. After 14 days, the tumors of the mice were removed and photographed, and the mass of these tumors was measured. In addition, we designed another set of experiments to test whether the recombinant SPARCL1 protein has an effect on subcutaneous tumors. NCG mice were injected subcutaneously with BCPAP cells. Ten days later, the mice were divided into two groups and treated by intratumoral injection of PBS or recombinant SPARCL1 protein. The volume of these tumors was measured using a vernier caliper during the experiment. After 21 days, the mouse tumors were removed and photographed, and the mass of these tumors was measured. For the lung metastasis mouse model, 5 × 10^5^ BCPAP-NC or BCPAP-SPARCL1 cells were injected into NCG mice via the tail vein to form tumors *in vivo*. After 28 days, the lungs, livers, and kidneys of the mice were removed and photographed, and the number of tumor nodules on the surface of these organs was counted.

### Statistical analysis

Statistical analysis was performed on the experimental data of the TPC-1 and BCPAP groups and their respective control groups using GraphPad 9.0 software, which involved *t*-test verification for statistical processing. Significance is represented as follows: * indicates a significant difference between the overexpression group and the control group (*P* < 0.05), **P < 0.01, ***P < 0.001, and ****P < 0.0001.

## Result

### SPARCL1 is lowly expressed in PTC, and its elevated expression is closely related to better prognosis

**Figure 1:**
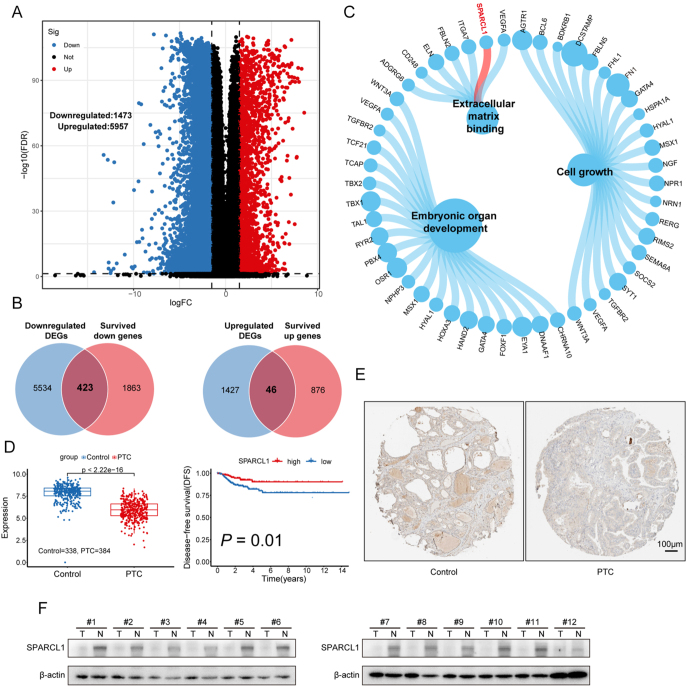
SPARCL1 is lowly expressed in PTC and associated with patient prognosis. (A) Volcano plot of RNA sequencing data from thyroid tissues of PTC patients and normal subjects in the TCGA database. (B) Venn diagram showing the intersection of differentially expressed genes and prognosis-related genes. (C) GO enrichment analysis of intersection genes in panel (B). (D) Boxplot of SPARCL1 mRNA expression in healthy people and PTC patients and Kaplan–Meier curve of disease-free survival for SPARCL1 in PTC patients. (E) Representative images of immunohistochemistry of SPARCL1 in healthy people and PTC patients from the HPA database. (F) The protein expression of SPARCL1 in thyroid tumor tissues and adjacent tissues from 12 PTC patients was detected by western blot. T represents the tumor tissue, and N represents the tissue adjacent to the tumor.

To screen for differentially expressed genes (DEGs) between PTC and normal thyroid tissues, we analyzed PTC RNA-seq data from the TCGA database and found that there were 1,473 upregulated and 5,957 downregulated DEGs in PTC compared to normal tissues (Fig. 1A). To identify genes associated with prognosis among these DEGs, we intersected them with a list of prognostically relevant genes and found that there were 423 downregulated and 46 upregulated intersection genes (Fig. 1B). Then, we performed GO enrichment analysis on these intersection genes and discovered that *extracellular matrix binding* ranked high among the enriched terms (Fig. 1C). The gene *SPARCL1* in that term has been reported to be a tumor suppressor gene, and the protein it encodes is a secreted protein, which attracted our attention (Fig. 1C). To verify the aberrant expression of SPARCL1, we analyzed the RNA-seq data and clinical information of PTC patients in the TCGA database and found that SPARCL1 is significantly downregulated in PTC and that patients with low SPARCL1 expression have a shorter disease-free survival (Fig. 1D). We also confirmed the reduced protein expression of SPARCL1 in PTC through immunohistochemistry in the Human Protein Atlas (HPA) database (Fig. 1E). Furthermore, results from clinical samples showed that the protein expression of SPARCL1 in tumor tissue was significantly lower than that in normal tissue adjacent to the tumor in PTC (Fig. 1F). Taken together, these results demonstrate that SPARCL1 acts as a tumor suppressor gene in PTC, suggesting its great potential as an innovative therapeutic agent for PTC.

### Overexpression of SPARCL1 inhibits the proliferation, migration, and invasion and promotes apoptosis of PTC cells

**Figure 2:**
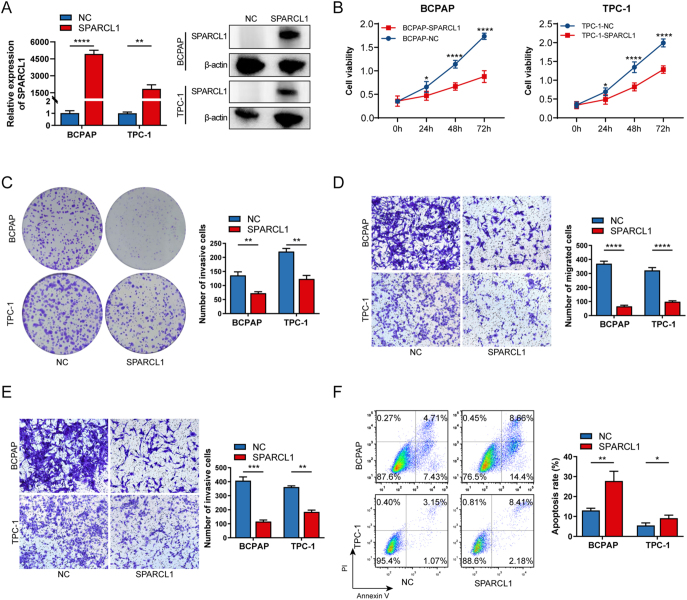
Overexpressing SPARCL1 inhibits proliferation, migration, and invasion and promotes apoptosis in PTC cells. (A) RT-qPCR and western blot analysis were used to verify whether SPARCL1 was successfully overexpressed in BCPAP and TPC-1 cells. (B) CCK-8 assay of PTC cells in the overexpressing SPARCL1 group vs the control group. (C) Colony formation assay of PTC cells in the overexpressing SPARCL1 group vs the control group. (D and E) Transwell migration (D) and invasion (E) assays of PTC cells in the overexpressing SPARCL1 group vs the control group. (F) Apoptosis assay of PTC cells in the overexpressing SPARCL1 group vs the control group. *P value <0.05; **P value <0.01; ***P value <0.001; ****P value <0.0001.

To determine whether SPARCL1 is a tumor suppressor gene in PTC, we first selected two PTC cell lines (including BCPAP cells and TPC-1 cells) for relevant phenotypic and mechanistic experiments. The results of RT-qPCR and western blot showed that compared with the control group, the mRNA and protein levels of SPARCL1 of the cells in the overexpression group were significantly increased, which indicated that PTC cells overexpressing SPARCL1 were successfully constructed (Fig. 2A and Supplementary Material 2). To explore the effect of overexpression of SPARCL1 on the proliferation ability of PTC cells, we performed CCK-8 and colony formation experiments. The results showed that the cell proliferation rate in the overexpression group was slower than that in the control group (Fig. 2B and C). What’s more, the results of the Transwell migration and invasion assay showed that the number of cells that migrated and invaded in the SPARCL1 overexpression group was significantly lower than that in the control group (Fig. 2D and E), which indicates that overexpression of SPARCL1 weakened the migration and invasion capabilities of BCPAP and TPC-1 cells. In addition, apoptosis experiments showed that compared with the control group, the PTC cells overexpressing SPARCL1 showed a higher apoptosis rate (Fig. 2F), which indicated that overexpression of SPARCL1 promoted the apoptosis of BCPAP and TPC-1 cells. From these results, it is evident that the SPARCL1 gene inhibits the carcinogenesis of PTC cells, which indicates that it plays a role as a tumor suppressor gene in the malignant behavior of PTC.

### The supernatant secreted by PTC-SPARCL1 cells suppresses the proliferation, migration, and invasion of PTC cells and promotes apoptosis

**Figure 3:**
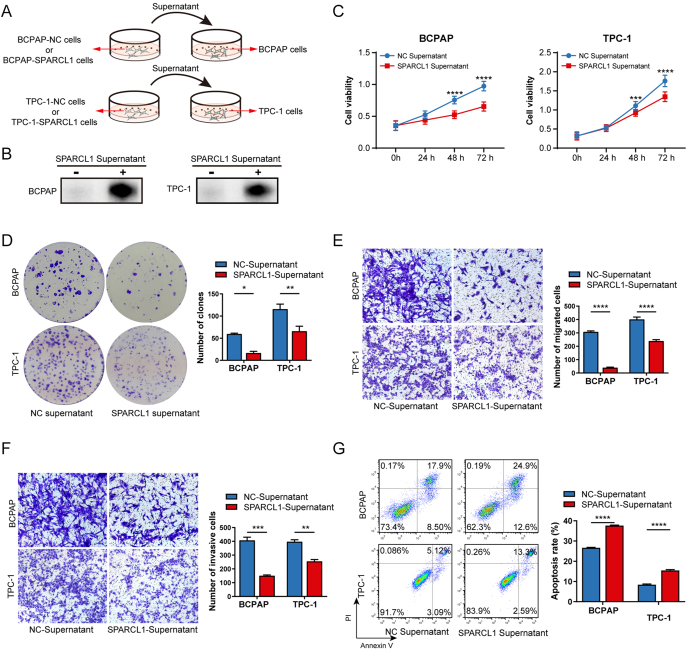
The SPARCL1 protein secreted by PTC-SPARCL1 cells suppresses the proliferation, migration, and invasion of PTC cells and promotes apoptosis. (A) Schematic representation of the conditioned medium transfer experiment. Conditioned medium from SPARCL1-overexpressing cells (BCPAP-SPARCL1 and TPC-1-SPARCL1) and control cells (PTC-NC) was collected, concentrated, and subsequently applied to wild-type PTC cell cultures. (B) The protein level of SPARCL1 in the collected supernatants was detected by western blot. (C) CCK-8 assays of PTC cells receiving supernatant secreted by PTC-SPARCL1 cells or PTC-NC cells. (D) Colony formation assays of PTC cells receiving supernatant secreted by PTC-SPARCL1 cells or PTC-NC cells. (E and F) Transwell invasion (E) and migration (F) assays of PTC cells receiving supernatant secreted by PTC-SPARCL1 cells or PTC-NC cells. (G) Apoptosis assay of PTC cells receiving supernatant secreted by PTC-SPARCL1 cells or PTC-NC cells. *P value <0.05; **P value <0.01; ***P value <0.001; ****P value <0.0001.

Considering that SPARCL1 is a secreted protein, we collected the supernatant of PTC cells infected with lentivirus and then used it to treat the corresponding normal PTC cell lines (in particular, BCPAP cells were treated with supernatant from BCPAP-SPARCL1/NC cells, and TPC-1 cells with supernatant from TPC-1-SPARCL1/NC cells) to observe changes in the function of these cells (Fig. 3A). Then, our results showed the presence of SPARCL1 protein in the supernatant secreted by PTC-SPARCL1 cells, whereas it was absent in the supernatant secreted by PTC-NC cells (Fig. 3B). The results of CCK-8 and colony formation assays demonstrated that the proliferation rate of PTC cells receiving supernatant secreted by PTC-SPARCL1 cells was significantly lower than that receiving supernatant secreted by PTC-NC cells (Fig. 3C and D). Moreover, Transwell migration and invasion assays showed that the number of migrating and invasive cells receiving supernatant secreted by PTC-SPARCL1 cells was significantly lower than that receiving supernatant secreted by PTC-NC cells (Fig. 3E and F), indicating that the SPARCL1 in the supernatant weakened the migration and invasion abilities of PTC cells. Furthermore, the results of apoptosis assays revealed a higher apoptotic rate in the PTC cells receiving supernatant secreted by PTC-SPARCL1 cells compared to the cells receiving supernatant secreted by PTC-NC cells (Fig. 3G), suggesting that the SPARCL1 in the supernatant promoted the apoptosis of PTC cells. In summary, our results suggest that SPARCL1 protein can be secreted outside the cell and then act on tumor cells to exert an inhibitory effect.

### Recombinant SPARCL1 protein suppresses the proliferation, migration, and invasion of PTC cells while promoting apoptosis

**Figure 4:**
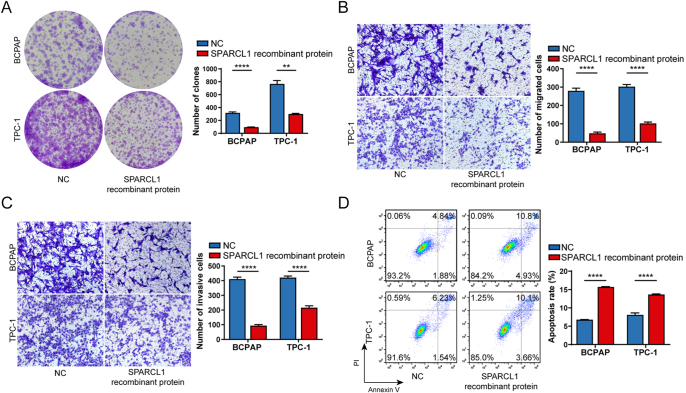
Recombinant SPARCL1 protein suppresses proliferation, migration, and invasion of PTC cells while promoting apoptosis. (A) Colony formation assays of PTC cells treated with recombinant SPARCL1 protein or PBS. (B and C) Transwell migration (B) and invasion (C) assays of PTC cells treated with recombinant SPARCL1 protein and PBS. (D) Apoptosis assay of PTC cells treated with recombinant SPARCL1 protein and PBS. **P value <0.01; ***P value <0.001; ****P value <0.0001.

To further investigate the potential of SPARCL1 as a novel therapeutic candidate for PTC, we employed recombinant SPARCL1 protein to evaluate its direct impact on PTC cells. The results of the CCK-8 assay showed that the proliferation rate of PTC cells treated with recombinant SPARCL1 protein was relatively slow compared with the control group (Fig. 4A). The results of Transwell assays showed that there were fewer migrated and invasive cells in the recombinant SPARCL1 protein group than in the control group (Fig. 4B and C). This suggests that recombinant SPARCL1 protein attenuates the migration and invasiveness of PTC cells. In addition, the results of the cell apoptosis experiment showed that there was a higher rate of apoptosis in the PTC cells treated with recombinant SPARCL1 protein (Fig. 4D) compared with the control group, implying that recombinant SPARCL1 protein promotes apoptosis in PTC cells. In summary, our results indicate that recombinant SPARCL1 protein, similar to the supernatant secreted by PTC-SPARCL1 cells, inhibits the malignant biological behaviors of PTC cells, highlighting its effectiveness as an additional or innovative therapeutic agent.

### SPARCL1 inhibits the formation of tumor tissue and the multi-organ metastasis of tumor cells *in vivo*

**Figure 5:**
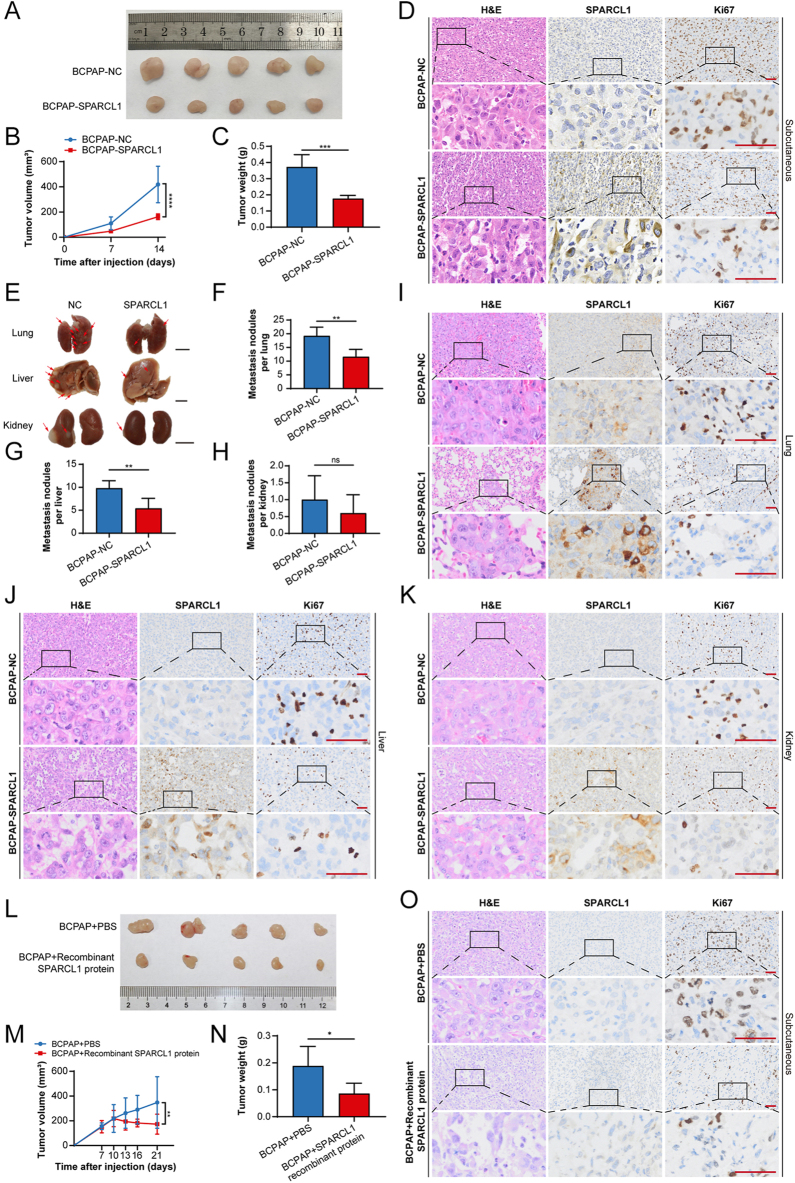
SPARCL1 inhibits the formation and multi-organ metastasis of tumor tissue *in vivo*. (A–D) BCPAP-NC and BCPAP-SPARCL1 cells were injected subcutaneously into NCG mice, and the volume of these tumors was measured using a vernier caliper (B). After 14 days, the tumors of the mice were removed and photographed (A) and the mass of these tumors was measured (C). The subcutaneous tumors of these mice were then prepared into paraffin sections and subjected to H&E staining and immunohistochemical staining for SPARCL1 and Ki67 (D). (E–K) BCPAP-NC and BCPAP-SPARCL1 cells were injected into NCG mice via the tail vein to form tumors *in vivo*. After 28 days, lungs (E and F), livers (E and G), and kidneys (E and H) of the mice were removed and photographed, and the number of tumor nodules on the surface of these organs was counted. The lungs (I), livers (J), and kidneys (K) of these mice were then prepared into paraffin sections and subjected to H&E staining and immunohistochemical staining for SPARCL1 and Ki67. (L–O) NCG mice were injected subcutaneously with BCPAP cells, and PBS or recombinant SPARCL1 protein was injected intratumorally 10 days later for treatment. The volume of these tumors was measured using a vernier caliper during the experiment (M). After 21 days, the mouse tumors were removed and photographed (L), and the mass of these tumors was measured (N). The subcutaneous tumors of these mice were then prepared into paraffin sections and subjected to H&E staining and immunohistochemical staining for SPARCL1 and Ki67 (O). The scale bar in panel (E) represents 5 mm, and the scale bar in panels (D, I, J, K, and O) represents 50 μm. *P value <0.05; **P value <0.01; ***P value <0.001; ****P value <0.0001; ns: no statistical difference.

To further explore the role of SPARCL1 in PTC, we conducted animal experiments *in vivo*. First, the results of the subcutaneous tumor formation experiment showed that compared with BCPAP-NC cells, the subcutaneous tumors formed by BCPAP-SPARCL1 cells were smaller in mass and volume (Fig. 5A, B, C). Immunohistochemistry results showed that overexpression of SPARCL1 reduced the expression of the proliferation marker Ki67 in subcutaneous tumors (Fig. 5D). After injecting BCPAP-NC cells or BCPAP-SPARCL1 cells into the tail vein of NCG mice, we found that these cells metastasized to the lungs, liver, and kidneys of mice (Fig. 5E). Compared with BCPAP-NC cells, BCPAP-SPARCL1 cells formed fewer tumor nodules in the lungs and liver of mice, indicating that SPARCL1 can inhibit tumor cell metastasis to multiple sites in PTC (Fig. 5F, G, H). Overexpression of SPARCL1 also reduced the expression of the proliferation marker Ki67 in tumor nodules metastasizing to the lung (Fig. 5I), liver (Fig. 5J), and kidney (Fig. 5K). To further explore the therapeutic potential of SPARCL1 in PTC, we established additional subcutaneous tumor models using BCPAP cells and treated these mice via intratumoral injection of recombinant SPARCL1 protein. The results showed that the subcutaneous tumors of mice treated with recombinant SPARCL1 protein were smaller in mass and volume (Fig. 5L, M, N), and the expression of the proliferation marker Ki67 in tumors was lower (Fig. 5O), indicating that recombinant SPARCL1 protein can inhibit tumor growth in PTC. Taken together, these results indicate that SPARCL1 suppresses tumor formation *in vivo*, reinforcing the efficacy of SPARCL1 restoration as an innovative therapeutic strategy.

### SPARCL1 inhibits the malignant biological behavior of PTC cells through the ferroptosis pathway

**Figure 6:**
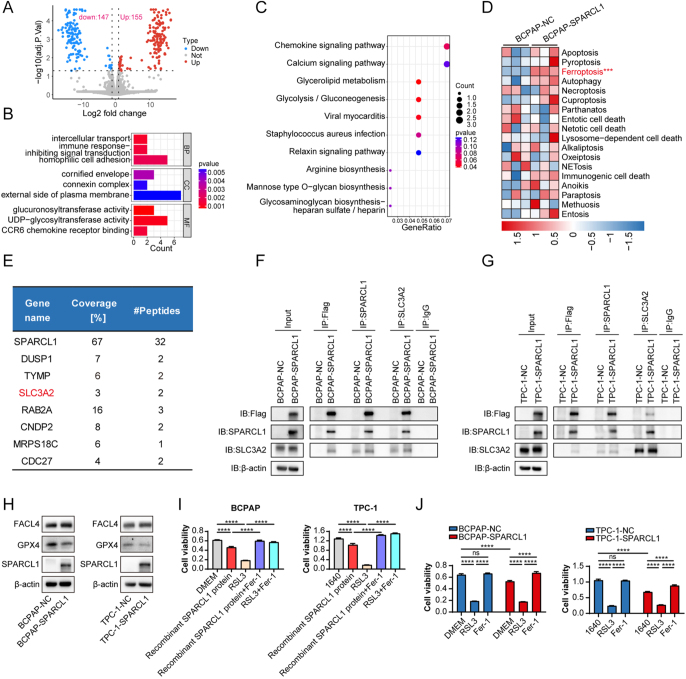
SPARCL1 inhibits the malignant biological behavior of PTC cells through the ferroptosis pathway. (A) Identification of differentially expressed genes (DEGs) between BCPAP-NC and BCPAP-SPARCL1 cells. The criteria for identifying DEGs were the adjusted *P* value < 0.05 and |log_2_ fold change|≥1. (B) GO enrichment analysis of DEGs, showing the top ten enriched items. (C) KEGG enrichment analysis of DEGs, displaying the top ten enriched items. (D) Gene set enrichment analysis (GSEA) of mRNA between BCPAP-SPARCL1 and BCPAP-NC cells based on the gene set of 18 cell death modes. (E) The top eight proteins interacting with SPARCL1 were identified by immunoprecipitation and mass spectrometry. (F and G) The interaction between SPARCL1 and SLC3A2 was verified by co-immunoprecipitation in BCPAP cells (F) and PTC cells (G). (H) The protein expression levels of FACL4 and GPX4 in PTC cells overexpressing SPARCL1 were detected by western blot. (I) BCPAP cells and TPC-1 cells were plated into 96-well plates and then treated with RSL3, Fer-1, and a certain amount of recombinant SPARCL1 protein on the next day. After 48 h, the cell activity of these cells was detected by the CCK-8 assay. (J) BCPAP-NC or BCPAP-SPARCL1 cells and TPC-1-NC cells or TPC-1-SPARCL1 cells were plated into 96-well plates and then treated with RSL3 and Fer-1 on the next day. After 48 h, the cell activity was detected by CCK-8 assay. ****P value <0.0001; ns: no statistical difference.

Next, to explore the specific mechanism by which SPARCL1 inhibits the carcinogenesis of PTC cells, we first performed mRNA-seq on BCPAP-SPARCL1 and BCPAP-NC cells. After identifying DEGs, we found there were 155 up-regulated genes and 147 down-regulated genes between them (Fig. 6A, Supplementary Material 2). Then, we performed functional enrichment analysis on DEGs to explore the biological pathways through which SPARCL1 functions in PTC. The results of GO enrichment analysis showed that the DEGs mediated by SPARCL1 mainly function through biological processes such as*homophilic cell adhesion* and *glucuronosyltransferase activity* (Fig. 6B). In addition, the results of KEGG enrichment analysis showed that the DEGs mediated by SPARCL1 mainly function through *glycerolipid metabolism*, *glycolysis/gluconeogenesis*, and other signaling pathways (Fig. 6C). Considering that gene set enrichment analysis (GSEA) is a functional enrichment analysis of all genes (not just differentially expressed genes), we ranked all genes based on the gene set of 18 cell death modes according to logFC, and then performed GSEA scoring and statistical analysis. We found that the ferroptosis score of BCPAP-SPARCL1 cells was significantly higher than that of BCPAP-NC cells (Fig. 6D).

Subsequently, we identified multiple proteins that interacted with SPARCL1 by immunoprecipitation and mass spectrometry (Fig. 6E), among which the solute carrier family 3 member 2 (SLC3A2) protein was a key protein in the ferroptosis pathway, which echoed the results of GSEA in mRNA-seq and aroused our interest. The interaction between SPARCL1 and SLC3A2 was confirmed by co-immunoprecipitation in two types of PTC cells (Fig. 6F and G). We then found that after overexpressing SPARCL1 in PTC cells, the key ferroptosis protein FACL4 was significantly upregulated, while GPX4 was significantly downregulated (Fig. 6H). RSL3, a GXP4 inhibitor, has been reported to induce ferroptosis in PTC (37). The results of the CCK-8 assay showed that RSL3 and recombinant SPARCL1 protein could reduce the cell viability of PTC cells, while treatment of these cells with Fer-1, a ferroptosis inhibitor, could restore the cell viability of PTC cells (Fig. 6I). Similarly, PTC cells overexpressing SPARCL1 showed reduced cell activity, while after these cells were treated with Fer-1, the cell activity of PTC-SPARCL1 cells was restored to a certain extent (Fig. 6J). These results suggest that SPARCL1 mediates ferroptosis of PTC cells through SLC3A2. In a word, SPARCL1 may inhibit the malignant biological behavior of PTC cells through the SLC3A2-mediated ferroptosis pathway.

## Discussion

It is reported that the lymph node metastasis rate of PTC patients is about 20–90%, which is the main reason for the high recurrence rate of PTC patients (38, 39). Therefore, exploring the causes of malignant proliferation and metastasis of tumor cells in PTC and finding therapeutic targets at the molecular level will be beneficial to the future treatment of PTC. Through bioinformatics analysis, we found that SPARCL1 may be involved in the invasion and metastasis of PTC as both a tumor suppressor and a secreted protein, suggesting that it may serve as a candidate for innovative restorative therapies in PTC. SPARCL1 is abnormally lowly expressed in PTC, and when it is lowly expressed, the prognosis of PTC patients is significantly worse. It has been reported that SPARCL1 also has similar expression patterns in breast cancer (33), skin melanoma (40), testicular germ cell tumors (40), and other cancers, revealing that SPARCL1 plays a tumor suppressor role in the occurrence and development of various tumors. However, the role and mechanism of SPARCL1 in PTC remain unclear.

Our cell experiments showed that overexpression of SPARCL1 inhibited the proliferation, migration, and invasion of PTC cells and promoted the apoptosis of PTC cells. Treating PTC cells with recombinant SPARCL1 protein alone also had a similar effect, which fully demonstrated the tumor suppressor role of SPARCL1 in PTC at the cellular level. Several studies have shown that overexpression of SPARCL1 can also inhibit the migration and invasion of prostate cancer cells (41, 42), renal cell carcinoma cells (43), colorectal cancer cells (44), and gastrointestinal stromal tumor cells (45), which indicates that SPARCL1 has the function of inhibiting the functions of various cancer cells.

Metastasis is the main cause of cancer-related death and the main reason for the high recurrence rate of PTC patients. The results of our experiments showed that overexpression of SPARCL1 in PTC cells can inhibit the growth of subcutaneous tumors in mice and inhibit the metastasis of PTC cells to the lungs, liver, and kidneys, which indicates that SPARCL1 also plays a tumor suppressor role *in vivo*. While the tail vein injection model classically results in pulmonary metastases due to the first-pass effect, we also observed metastatic nodules in the liver and kidneys. This extrapulmonary dissemination suggests that a subset of these PTC cells possesses exceptional invasive capabilities, allowing them to escape the pulmonary microcirculation and colonize distant, highly vascularized organs via the systemic circulation (46, 47). This further underscores the aggressive nature of the control cells and the robust inhibitory effect of SPARCL1. In colorectal cancer (44) and gastrointestinal stromal tumors (45), overexpression of SPARCL1 can also inhibit tumor growth and liver metastasis in mouse xenograft models. It has been reported that SPARCL1 has an anti-angiogenic effect, which may be the reason why SPARCL1 inhibits PTC cell metastasis (48, 49). In our research results, the growth of subcutaneous tumors formed by PTC cells was significantly inhibited after treatment with recombinant SPARCL1 protein, which suggests that recombinant SPARCL1 protein holds promise as a potential therapeutic agent for the treatment of PTC.

**Figure 7:**
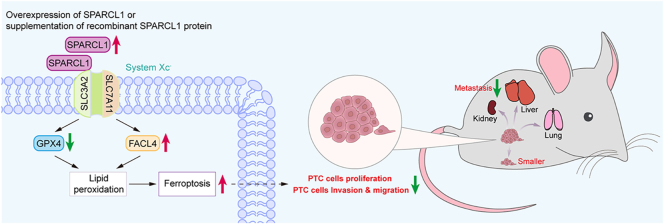
Mechanism diagram of this study. Overexpression of SPARCL1 or supplementation of recombinant SPARCL1 protein promotes the activation of the SLC3A2-mediated ferroptosis pathway and inhibits the proliferation, invasion, and migration of PTC cells, ultimately leading to the inhibition of tumor growth and multi-organ metastasis of PTC cells *in vivo*.

Mechanistically, we identified multiple DEGs by RNA-seq, which may function through pathways such as the Jak-STAT signaling pathway. Currently, many studies have reported the role of the Jak-STAT signaling pathway in PTC (50, 51, 52). For example, transducin-like enhancement of split 4 (TLE4) can inhibit tumor growth and metastasis through the JAK/STAT pathway in PTC (50). In addition, through the joint analysis of RNA-seq and interacting proteins, we focused on ferroptosis and SLC3A2. SLC3A2 is mainly located on the cell membrane. Related studies have shown that SLC3A2 is closely related to the intracellular redox reaction, and its dysregulation can induce ferroptosis in cells (53). Ferroptosis is a type of cell death driven by iron-dependent lipid peroxidation (54, 55). FACL4 (also known as ACSL4, acyl-CoA synthetase long-chain family member 4) is the first gene discovered to promote ferroptosis (56). FACL4 can catalyze the esterification reaction between polyunsaturated fatty acids (PUFAs) and acetyl coenzyme A (CoA) to form esters. The newly generated esters will further undergo peroxidation reactions, ultimately causing ferroptosis (57, 58, 59). Glutathione peroxidase 4 (GPX4) can use glutathione (GSH) to reduce phospholipid hydroperoxides to phospholipid alcohols, thereby inhibiting the occurrence of ferroptosis (60, 61). System Xc^−^ and GPX4 usually work together as strong inhibitors of ferroptosis. System Xc^−^ is an amino acid transporter on the cell membrane (composed of SLC7A11 and SLC3A2), which functions to import cystine into cells and export glutamate, promoting GSH synthesis (62). Once system Xc^−^ is inhibited (e.g. by downregulating SLC7A11 (63) and SLC3A2 (64)), it will lead to a lack of antioxidants in the cell, thereby promoting the occurrence of ferroptosis (65). Some studies have reported that downregulation of SLC7A11 promotes ferroptosis in PTC (21, 66), but no reports on SLC3A2 have been seen in PTC. We confirmed the interaction between SPARCL1 and SLC3A2 and found that SPARCL1 can affect the expression of ferroptosis key proteins FACL4 and GPX4. At the same time, our further experiments showed that overexpression of SPARCL1 or addition of recombinant SPARCL1 protein in PTC cells can reduce the activity of PTC cells, and this phenomenon can be reversed by ferroptosis inhibitors. These results fully demonstrate that SPARCL1 induces ferroptosis in PTC (Fig. 7).

Our study also has some limitations. On the one hand, we did not knock down SPARCL1 in PTC cells to study its effect on PTC cells, mainly because we considered that SPARCL1 is expressed at a low level in PTC cells and is, therefore, not suitable for knockdown or knockout experiments. On the other hand, the detailed mechanism of how SPARCL1 specifically promotes ferroptosis in PTC needs further study. In conclusion, our findings highlight the great potential of SPARCL1 as a novel therapeutic candidate to significantly suppress the malignant progression of PTC. In light of this, therapeutic interventions that enhance SPARCL1 function may represent a promising strategy for the treatment of PTC.

## Supplementary materials





## Declaration of interest

The authors declare that they have no known competing financial interests or personal relationships that could have influenced the work reported in this paper. The authors declare no conflicts of interest.

## Funding

This work was supported by the Project of Sichuan Clinical Research Center for Radiation and Therapy, Key Laboratory of Nuclear and Radiation Damage Mechanisms and Treatment Technologies at Chengdu Medical College of Sichuan Province, the Second Affiliated Hospital of Chengdu Medical College, Nuclear Industry 416 Hospital (24LHHYFS-05, 24LHHYFS-08, and 2024ZX02), Scientific Research Project of Medicine Department of Chengdu City (2024308), Special Project of ‘Technological Innovation’ Project of CNNC Medical Industry Co. Ltd (ZHYLZD2025012), the Foundation of Key Laboratory of Radiation Physics and Technology of the Ministry of Education (2023SCURPT03), and Medical Youth Innovation Project of Sichuan Province (Q22060).

## Author contribution statement

FW, JZ, XZ, YG, LR, and HY performed the experiments and drafted the manuscript. DX, SC, and QL assisted in performing part of the experiments and analyzed the data. FW and DX formatted and modified the figures in this research. WT, KZ, XX, and ZJ performed the bioinformatics analysis. ZJ, YS, and WT designed the research and revised the manuscript. All authors have read and approved the final manuscript.

## Statement of ethics

An ethics statement is not applicable as this study is based exclusively on the published literature.

## References

[1] Cabanillas ME, McFadden DG & Durante C. Thyroid cancer. Lancet 2016 388 2783–2795. (10.1016/S0140-6736(16)30172-6) 27240885

[2] Fagin JA & Wells SA. Biologic and clinical perspectives on thyroid cancer. N Engl J Med 2016 375 1054–1067. (10.1056/NEJMra1501993) 27626519 PMC5512163

[3] Haugen BR, Alexander EK, Bible KC, et al. 2015 American Thyroid Association Management Guidelines for adult patients with thyroid nodules and differentiated thyroid cancer: the American Thyroid Association Guidelines task force on thyroid nodules and differentiated thyroid cancer. Thyroid 2016 26 1–133. (10.1089/thy.2015.0020) 26462967 PMC4739132

[4] Kang IK, Kim K, Park J, et al. Central lymph node ratio predicts recurrence in patients with N1b papillary thyroid carcinoma. Cancers 2022 14 3677. (10.3390/cancers14153677) 35954338 PMC9367408

[5] Wang Z, Song X, Wang T, et al. Defining the ATA-2025 “consider RAIT” zone in older patients with N1b PTC. Endocr Relat Cancer 2026 33 e250420. (10.1530/ERC-25-0420) 41543025 PMC12910568

[6] Rajabi S, Alix-Panabières C, Alaei AS, et al. Looking at thyroid cancer from the tumor-suppressor genes point of view. Cancers 2022 14 2461. (10.3390/cancers14102461) 35626065 PMC9139614

[7] Lee EYHP & Muller WJ. Oncogenes and tumor suppressor genes. Cold Spring Harb Perspect Biol 2010 2 a003236. (10.1101/cshperspect.a003236) 20719876 PMC2944361

[8] Dakal TC, Dhabhai B, Pant A, et al. Oncogenes and tumor suppressor genes: functions and roles in cancers. MedComm 2024 5 e582. (10.1002/mco2.582) 38827026 PMC11141506

[9] Maziveyi M & Alahari SK. Breast cancer tumor suppressors: a special emphasis on novel protein nischarin. Cancer Res 2015 75 4252–4259. (10.1158/0008-5472.CAN-15-1395) 26392073

[10] Wu F, Liu Q, Zhang J, et al. Prolyl 4-hydroxylase subunit alpha-2 acts as a TRIM21 ubiquitination substrate to promote papillary thyroid cancer progression via the glycolytic pathway. Cell Death Dis 2025 16 395. (10.1038/s41419-025-07702-0) 40379610 PMC12084645

[11] Zou M, Baitei EY, Al-Rijjal RA, et al. TSH overcomes Braf(V600E)-induced senescence to promote tumor progression via downregulation of p53 expression in papillary thyroid cancer. Oncogene 2016 35 1909–1918. (10.1038/onc.2015.253) 26477313 PMC6310059

[12] Wójcicka A, Czetwertyńska M, Świerniak M, et al. Variants in the ATM-CHEK2-BRCA1 axis determine genetic predisposition and clinical presentation of papillary thyroid carcinoma. Genes Chromosomes Cancer 2014 53 516–523. (10.1002/gcc.22162) 24599715 PMC4058861

[13] Liu D, Yang C, Bojdani E, et al. Identification of RASAL1 as a major tumor suppressor gene in thyroid cancer. J Natl Cancer Inst 2013 105 1617–1627. (10.1093/jnci/djt249) 24136889 PMC3818169

[14] Alvarez-Nuñez F, Bussaglia E, Mauricio D, et al. PTEN promoter methylation in sporadic thyroid carcinomas. Thyroid 2006 16 17–23. (10.1089/thy.2006.16.17) 16487009

[15] Prasad V. Perspective: the precision-oncology illusion. Nature 2016 537 S63. (10.1038/537S63a) 27602743

[16] Tannock IF & Hickman JA. Limits to personalized cancer medicine. N Engl J Med 2016 375 1289–1294. (10.1056/NEJMsb1607705) 27682039

[17] Song H, Wu J, Tang Y, et al. Diverse rescue potencies of p53 mutations to ATO are predetermined by intrinsic mutational properties. Sci Transl Med 2023 15 eabn9155. (10.1126/scitranslmed.abn9155) 37018419

[18] Wu S, Zhu J, Jiang T, et al. Long non-coding RNA ACTA2-AS1 suppresses metastasis of papillary thyroid cancer via regulation of miR-4428/KLF9 axis. Clin Epigenetics 2024 16 10. (10.1186/s13148-023-01622-6) 38195623 PMC10775490

[19] Yin D, Wang K, Zhao J, et al. IPCEF1: expression patterns, clinical correlates and new target of papillary thyroid carcinoma. J Cancer 2024 15 6434–6451. (10.7150/jca.98470) 39513122 PMC11540494

[20] Gou Q, Gao L, Nie X, et al. Long noncoding RNA AB074169 inhibits cell proliferation via modulation of KHSRP-mediated CDKN1a expression in papillary thyroid carcinoma. Cancer Res 2018 78 4163–4174. (10.1158/0008-5472.CAN-17-3766) 29735546

[21] Ji F-H, Fu X-H, Li G-Q, et al. FTO prevents thyroid cancer progression by SLC7A11 m6A methylation in a ferroptosis-dependent manner. Front Endocrinol 2022 13 857765. (10.3389/fendo.2022.857765) PMC9205202 35721711

[22] Liu B, Xiang L, Ji J, et al. Sparcl1 promotes nonalcoholic steatohepatitis progression in mice through upregulation of CCL2. J Clin Investig 2021 131 e144801. (10.1172/JCI144801) 34651580 PMC8516465

[23] Miao Y, Wu S, Gong Z, et al. SPARCL1 promotes chondrocytes extracellular matrix degradation and inflammation in osteoarthritis via TNF/NF-κB pathway. J Orthop Translat 2024 46 116–128. (10.1016/j.jot.2024.02.009) 38867741 PMC11167206

[24] Cheng X, Chen X, Zhang M, et al. Sparcl1 and atherosclerosis. J Inflamm Res 2023 16 2121–2127. (10.2147/JIR.S406907) 37220502 PMC10200116

[25] Girard JP & Springer TA. Cloning from purified high endothelial venule cells of hevin, a close relative of the antiadhesive extracellular matrix protein SPARC. Immunity 1995 2 113–123. (10.1016/1074-7613(95)90083-7) 7600298

[26] Shen C, Han L, Liu B, et al. The KDM6A-SPARCL1 axis blocks metastasis and regulates the tumour microenvironment of gastrointestinal stromal tumours by inhibiting the nuclear translocation of p65. Br J Cancer 2022 126 1457–1469. (10.1038/s41416-022-01728-3) 35136209 PMC9090789

[27] Isler SG, Schenk S, Bendik I, et al. Genomic organization and chromosomal mapping of SPARC-like 1, a gene down regulated in cancers. Int J Oncol 2001 18 521–526. (10.3892/ijo.18.3.521) 11179481

[28] Petersen I, Bujard M, Petersen S, et al. Patterns of chromosomal imbalances in adenocarcinoma and squamous cell carcinoma of the lung. Cancer Res 1997 57 2331–2335. 9192802

[29] Shivapurkar N, Virmani AK, Wistuba II, et al. Deletions of chromosome 4 at multiple sites are frequent in malignant mesothelioma and small cell lung carcinoma. Clin Cancer Res 1999 5 17–23. 9918198

[30] Schwendel A, Richard F, Langreck H, et al. Chromosome alterations in breast carcinomas: frequent involvement of DNA losses including chromosomes 4q and 21q. Br J Cancer 1998 78 806–811. (10.1038/bjc.1998.583) 9743305 PMC2062965

[31] Rosin MP, Cairns P, Epstein JI, et al. Partial allelotype of carcinoma in situ of the human bladder. Cancer Res 1995 55 5213–5216. 7585577

[32] Califano JA, Johns MM, Westra WH, et al. An allelotype of papillary thyroid cancer. Int J Cancer 1996 69 442–444. (10.1002/(SICI)1097-0215(19961220)69:6<442::AID-IJC3>3.0.CO;2-4) 8980243

[33] Chen M, Zheng W & Fang L. Identifying liver metastasis-related hub genes in breast cancer and characterizing SPARCL1 as a potential prognostic biomarker. PeerJ 2023 11 e15311. (10.7717/peerj.15311) 37180578 PMC10174054

[34] Zhang S, Zhang F & Feng L. The inhibition of HeLa cells proliferation through SPARCL1 mediated by SPP1. Cytotechnology 2021 73 71–78. (10.1007/s10616-020-00443-2) 33505115 PMC7817754

[35] Hurley PJ, Hughes RM, Simons BW, et al. Androgen-regulated SPARCL1 in the tumor microenvironment inhibits metastatic progression. Cancer Res 2015 75 4322–4334. (10.1158/0008-5472.CAN-15-0024) 26294211 PMC4609262

[36] Wu F, Zhang X, Zhang S, et al. Construction of an immune-related lncRNA-miRNA-mRNA regulatory network in radiation-induced esophageal injury in rats. Int Immunopharmacol 2023 122 110606. (10.1016/j.intimp.2023.110606) 37423154

[37] Sekhar KR, Hanna DN, Cyr S, et al. Glutathione peroxidase 4 inhibition induces ferroptosis and mTOR pathway suppression in thyroid cancer. Sci Rep 2022 12 19396. (10.1038/s41598-022-23906-2) 36371529 PMC9653479

[38] Xu X, Li C, Yu X, et al. Clinicopathological features affecting the efficacy in 131I ablation therapy of papillary thyroid carcinoma with lymph node metastasis. Front Endocrinol 2024 15 1382009. (10.3389/fendo.2024.1382009) PMC11288842 39086895

[39] Podnos YD, Smith D, Wagman LD, et al. The implication of lymph node metastasis on survival in patients with well-differentiated thyroid cancer. Am Surg 2005 71 731–734. (10.1177/000313480507100907) 16468507

[40] He K, Li C, Yuan H, et al. Immunological role and prognostic value of SPARCL1 in pan-cancer analysis. Pathol Oncol Res 2022 28 1610687. (10.3389/pore.2022.1610687) 36483097 PMC9722748

[41] Xiang Y, Qiu Q, Jiang M, et al. SPARCL1 suppresses metastasis in prostate cancer. Mol Oncol 2013 7 1019–1030. (10.1016/j.molonc.2013.07.008) 23916135 PMC3838491

[42] Hurley PJ, Marchionni L, Simons BW, et al. Secreted protein, acidic and rich in cysteine-like 1 (SPARCL1) is down regulated in aggressive prostate cancers and is prognostic for poor clinical outcome. Proc Natl Acad Sci U S A 2012 109 14977–14982. (10.1073/pnas.1203525109) 22927397 PMC3443123

[43] Ye H, Wang W-G, Cao J, et al. SPARCL1 suppresses cell migration and invasion in renal cell carcinoma. Mol Med Rep 2017 16 7784–7790. (10.3892/mmr.2017.7535) 28944877

[44] Hu H, Zhang H, Ge W, et al. Secreted protein acidic and rich in cysteines-like 1 suppresses aggressiveness and predicts better survival in colorectal cancers. Clin Cancer Res 2012 18 5438–5448. (10.1158/1078-0432.CCR-12-0124) 22891198

[45] Shen C, Yin Y, Chen H, et al. Secreted protein acidic and rich in cysteine-like 1 suppresses metastasis in gastric stromal tumors. BMC Gastroenterol 2018 18 105. (10.1186/s12876-018-0833-8) 29973149 PMC6030747

[46] Huang Z-J, Li Y-J, Yang J, et al. PTPLAD1 regulates PHB-Raf interaction to orchestrate epithelial-mesenchymal and Mitofusion-Fission transitions in colorectal cancer. Int J Biol Sci 2024 20 2202–2218. (10.7150/ijbs.82361) 38617530 PMC11008263

[47] Deng S, Krutilina RI, Wang Q, et al. An orally available tubulin inhibitor, VERU-111, suppresses triple-negative breast cancer tumor growth and metastasis and bypasses taxane resistance. Mol Cancer Ther 2020 19 348–363. (10.1158/1535-7163.MCT-19-0536) 31645441 PMC7007836

[48] Lau CP-Y, Poon RT-P, Cheung S-T, et al. SPARC and Hevin expression correlate with tumour angiogenesis in hepatocellular carcinoma. J Pathol 2006 210 459–468. (10.1002/path.2068) 17029219

[49] Regensburger D, Tenkerian C, Pürzer V, et al. Matricellular protein SPARCL1 regulates blood vessel integrity and antagonizes inflammatory bowel disease. Inflamm Bowel Dis 2021 27 1491–1502. (10.1093/ibd/izaa346) 33393634 PMC8376124

[50] Lin J, Cai B, Lin Q, et al. TLE4 downregulation identified by WGCNA and machine learning algorithm promotes papillary thyroid carcinoma progression via activating JAK/STAT pathway. J Cancer 2024 15 4759–4776. (10.7150/jca.95501) 39006072 PMC11242334

[51] Dong Y, Tan H, Wang L, et al. Progranulin promoted the proliferation, metastasis, and suppressed apoptosis via JAK2-STAT3/4 signaling pathway in papillary thyroid carcinoma. Cancer Cell Int 2023 23 191. (10.1186/s12935-023-03033-2) 37660003 PMC10475200

[52] Couto JP, Daly L, Almeida A, et al. STAT3 negatively regulates thyroid tumorigenesis. Proc Natl Acad Sci U S A 2012 109 E2361–E2370. (10.1073/pnas.1201232109) 22891351 PMC3435219

[53] Chen Z, Zhang J, Gao S, et al. Suppression of Skp2 contributes to sepsis-induced acute lung injury by enhancing ferroptosis through the ubiquitination of SLC3A2. Cell Mol Life Sci 2024 81 325. (10.1007/s00018-024-05348-3) 39079969 PMC11335248

[54] Stockwell BR. Ferroptosis turns 10: emerging mechanisms, physiological functions, and therapeutic applications. Cell 2022 185 2401–2421. (10.1016/j.cell.2022.06.003) 35803244 PMC9273022

[55] Han M, Li S, Fan H, et al. Regulated cell death in glioma: promising targets for natural small-molecule compounds. Front Oncol 2024 14 1273841. (10.3389/fonc.2024.1273841) 38304870 PMC10830839

[56] Doll S, Proneth B, Tyurina YY, et al. ACSL4 dictates ferroptosis sensitivity by shaping cellular lipid composition. Nat Chem Biol 2017 13 91–98. (10.1038/nchembio.2239) 27842070 PMC5610546

[57] Küch E-M, Vellaramkalayil R, Zhang I, et al. Differentially localized acyl-CoA synthetase 4 isoenzymes mediate the metabolic channeling of fatty acids towards phosphatidylinositol. Biochim Biophys Acta 2014 1841 227–239. (10.1016/j.bbalip.2013.10.018) 24201376

[58] Soupene E & Kuypers FA. Mammalian long-chain Acyl-CoA synthetases. Exp Biol Med 2008 233 507–521. (10.3181/0710-MR-287) PMC3377585 18375835

[59] Yang Y, Zhu T, Wang X, et al. ACSL3 and ACSL4, distinct roles in ferroptosis and cancers. Cancers 2022 14 5896. (10.3390/cancers14235896) 36497375 PMC9739553

[60] Seiler A, Schneider M, Förster H, et al. Glutathione peroxidase 4 senses and translates oxidative stress into 12/15-lipoxygenase dependent- and AIF-mediated cell death. Cell Metab 2008 8 237–248. (10.1016/j.cmet.2008.07.005) 18762024

[61] Dixon SJ, Patel DN, Welsch M, et al. Pharmacological inhibition of cystine–glutamate exchange induces endoplasmic reticulum stress and ferroptosis. Elife 2014 3 e02523. (10.7554/eLife.02523) 24844246 PMC4054777

[62] Rochette L & Vergely C. Coronary artery disease: can aminothiols be distinguished from reactive oxygen species? Nat Rev Cardiol 2016 13 128–130. (10.1038/nrcardio.2016.20) 26864913

[63] Wang W, Green M, Choi JE, et al. CD8+ T cells regulate tumour ferroptosis during cancer immunotherapy. Nature 2019 569 270–274. (10.1038/s41586-019-1170-y) 31043744 PMC6533917

[64] Xiang P, Chen Q, Chen L, et al. Metabolite Neu5Ac triggers SLC3A2 degradation promoting vascular endothelial ferroptosis and aggravates atherosclerosis progression in ApoE−/−mice. Theranostics 2023 13 4993–5016. (10.7150/thno.87968) 37771765 PMC10526676

[65] Belalcázar AD, Ball JG, Frost LM, et al. Transsulfuration is a significant source of sulfur for glutathione production in human mammary epithelial cells. ISRN Biochem 2014 2013 637897. (10.1155/2013/637897) 24634789 PMC3949734

[66] Wang L, Zhang Y, Yang J, et al. The knockdown of ETV4 inhibits the papillary thyroid cancer development by promoting ferroptosis upon SLC7A11 downregulation. DNA Cell Biol 2021 40 1211–1221. (10.1089/dna.2021.0216) 34283663

