# Supplementary material for: The secreted protein SPARCL1 suppresses tumor progression in papillary thyroid carcinoma via SLC3A2-mediated ferroptosis
Source: Endocr Relat Cancer. 2026 Jun 8;33(6):e260018. doi: 10.1530/ERC-26-0018 (PMC13250643; doi:10.1530/ERC-26-0018)
Supplement: Supplementary file 1 [file supplementary_tables.pdf]

Table S1. Primer sequences.

		Primer sequence (5'-3')
GAPDH	Forward primer	ATCACCATCTTCCAGGAGCGA
	Reverse primer	CCTTCTCCATGGTGGTGAAGAC
SPARCL1	Forward primer	AAACGGTAGCACCTGACAAC
	Reverse primer	CTTATGGTGGGAATCGTCTT

Table S2. Cycle threshold (CT) values of RT-qPCR.

Sample	GAPDH	SPARCL1
BCPAP-NC-1	11.1	25.89
BCPAP-NC-2	11.84	25.99
BCPAP-NC-3	13	25.49
BCPAP-SPARC1-1	10.81	13.98
BCPAP-SPARC1-2	13.19	13.89
BCPAP-SPARC1-3	13.03	13.79
TPC-1-NC-1	12.66	27.38
TPC-1-NC-2	10.82	27.51
TPC-1-NC-3	13.12	27.67
TPC-1-SPARC1-1	10.94	16.66
TPC-1-SPARC1-2	11.43	16.26
TPC-1-SPARC1-3	13.15	16.08

Table S3. Gene set of 18 cell death modes.

Apoptosis	AATF、ABL1、ACAA2、ACKR3、ACVR1、ACVR1B、ADORA1、AEN、AGT、AGTR2、AIFM1、AKT1、ANXA6、APAF1、APPL1、AR、ARHGEF2、ARL6IP5、ARMC10、ARRB2、ASAH2、ATF3、ATF4、ATM、ATP2A1、ATP2A3、ATP5IF1、AVP、BAD、BAG3、BAG5、BAG6、BAK1、BAX、BBC3、BCAP31、BCL10、BCL2、BCL2A1、BCL2L1、BCL2L10、BCL2L11、BCL2L12、BCL2L14、BCL2L2、BCL3、BCLAF1、BDKRB2、BDNF、BECN1、BID、BIK、BIRC6、BLOC1S2、BMF、BMP4、BMP5、BMPR1B、BNIP3、BNIP3L、BOK、BRCA1、BRCA2、BRSK2、BTK、CAAP1、CASP1、CASP10、CASP12、CASP2、CASP3、CASP4、CASP5、CASP8、CASP8AP2、CASP9、CAV1、CCAR2、CCK、CD14、CD24、CD27、CD28、CD38、CD3E、CD44、CD5、CD70、CD74、CDIP1、CDKN1A、CDKN2D、CEBPB、CFLAR、CHAC1、CHCHD10、CHEK2、CIB1、CIDEB、CLU、COA8、COL2A1、CRADD、CREB3、CREB3L1、CRH、CRIP1、CSF2、CSNK2A1、CSNK2A2、CTH、CTNNA1、CTSC、CTTN、CUL1、CUL2、CUL3、CUL4A、CUL5、CX3CL1、CX3CR1、CXCL12、

	CYLD、CYP1B1、DAB2IP、DAP、DAP3、DAPK1、DAPK2、DAPK3、DAPL1、DAXX、DBH、DCC、DDIAS、DDIT3、DDIT4、DDX3X、DDX47、DDX5、DEDD、DEDD2、DELE1、DEPTOR、DIABLO、DIDO1、DNAJA1、DNAJC10、DNM1L、DPF2、DYRK2、E2F1、E2F2、EDA2R、EIF2AK3、ELL3、ENO1、EP300、EPHA2、EPO、ERBB3、ERCC6、ERN1、ERN2、ERO1A、ERP29、EYA1、EYA2、EYA3、EYA4、FADD、FAF1、FAIM、FAIM2、FAM162A、FAS、FASLG、FASTK、FBH1、FBXW7、FEM1B、FGA、FGB、FGF10、FGFR1、FGFR3、FGG、FHIT、FIGNL1、FIS1、FNIP2、FXN、FYN、FZD9、G0S2、GABARAP、GATA1、GATA4、GCLM、GDNF、GFRAL、GGCT、GHITM、GNAI2、GNAI3、GPER1、GPX1、GRINA、GSDME、GSK3A、GSK3B、GSKIP、GSTP1、GZMB、HDAC1、HERPUD1、HGF、HIC1、HIF1A、HINT1、HIP1、HIP1R、HIPK1、HIPK2、HMGB2、HMOX1、HNRNPK、HRAS、HRK、HSPA1A、HSPA1B、HSPB1、HTRA2、HTT、HYAL2、HYOU1、ICAM1、IFI16、IFI27、IFI27L1、IFI27L2、IFI6、IFNB1、IFNG、IGF1、IKBKE、IL12A、IL19、IL1A、IL1B、IL2、IL20RA、IL33、IL4、IL6R、IL7、INCA1、ING2、ING5、INHBA、INHBB、INS、ITGA6、ITGAM、ITGAV、ITM2C、ITPR1、ITPRIP、IVNS1ABP、JAK2、JMY、JUN、KDM1A、KITLG、KRT18、KRT8、LCK、LGALS12、LGALS3、LRRK2、LTBR、LY96、MADD、MAEL、MAGEA3、MAP2K5、MAP3K5、MAPK7、MAPK8、MAPK8IP1、MAPK8IP2、MAPK9、MARCHF7、MAZ、MCL1、MDM2、MELK、MFF、MIF、MIR132、MIR15A、MIR16-1、MIR17、MIR198、MIR21、MIR210、MIR221、MIR222、MIR26B、MIR27B、MIR449A、MKNK2、MLH1、MLLT11、MMP9、MNT、MOAP1、MPV17L、MSH2、MSH6、MSX1、MUC1、MUL1、MYBBP1A、NACC2、NANOS3、NBN、NCK1、NCK2、NDUFA13、NDUFS3、NFATC4、NFE2L2、NGF、NGFR、NKX3-1、NLE1、NME5、NMT1、NOC2L、NOG、NOL3、NONO、NOS3、NOX1、NR4A2、NUPR1、OPA1、P2RX4、P2RX7、P4HB、PAK2、PAK5、PARK7、PARP1、PARP2、PAWR、PCGF2、PDCD10、PDCD5、PDCD6、PDIA3、PDK1、PDK2、PDPK1、PDX1、PEA15、PELI3、PERP、PF4、PHIP、PHLDA3、PIAS4、PIDD1、PIH1D1、PIK3R1、PINK1、PLAGL2、PLAUR、PLEKHF1、PLSCR3、PMAIP1、PML、POLB、POU4F1、POU4F2、PPARD、PPIA、PPIF、PPM1F、PPP1CA、PPP1R13B、PPP1R15A、PPP2R1B、PPP3CC、PPP3R1、PRDX2、PRELID1、PRKCA、PRKCD、PRKDC、PRKN、PRKRA、PRODH、PSEN1、PSMD10、PSME3、PTEN、PTGIS、PTH、PTPMT1、PTPN1、PTPN2、PTPRC、PTTG1IP、PYCARD、QARS1、RACK1、RAF1、RB1、RB1CC1、RBCK1、RELA、RET、RFFL、RHOT1、RHOT2、RIPK1、RIPK3、RNF183、RNF186、RNF34、RNF41、RPL11、RPL26、RPS27L、RPS3、RPS6KB1、RPS7、RRP8、RTKN2、

	RTL10、S100A8、S100A9、SCG2、SCN2A、SCRT2、SELENOK、SELENOS、SENP1、SEPTIN4、SERINC3、SERPINE1、SFN、SFPQ、SFRP1、SFRP2、SGMS1、SGPL1、SGPP1、SH3RF1、SHH、SHISA5、SIAH1、SIAH2、SIRT1、SIVA1、SKIL、SLC25A5、SLC35F6、SLC9A3R1、SMAD3、SNAI1、SNAI2、SNW1、SOD1、SOD2、SORT1、SP100、SRC、SRPX、SST、SSTR3、ST20、STK11、STK24、STK25、STK3、STK4、STRADB、STX4、STYXL1、SYVN1、TAF9、TAF9B、TCF7L2、TERT、TFDP1、TFDP2、TFPT、TGFB1、TGFB2、TGFBR1、THBS1、TICAM1、TICAM2、TIMM50、TIMP3、TLR3、TLR4、TM2D1、TMBIM1、TMBIM6、TMC8、TMEM102、TMEM109、TMEM117、TMEM14A、TMEM161A、TNF、TNFAIP3、TNFRSF10A、TNFRSF10B、TNFRSF10C、TNFRSF12A、TNFRSF1A、TNFRSF1B、TNFRSF25、TNFSF10、TNFSF12、TOPORS、TP53、TP53BP2、TP63、TP73、TPD52L1、TPT1、TRADD、TRAF1、TRAF2、TRAF7、TRAP1、TRIAP1、TRIB3、TRIM32、TRIM39、TXNDC12、TYROBP、UACA、UBB、UBE2K、UBE4B、UBQLN1、UMOD、UNC5B、URI1、USP28、USP47、VDAC2、VNN1、WDR35、WNT4、WWOX、XBP1、YAP1、YBX3、YWHAB、YWHAE、YWHAG、YWHAH、YWHAQ、YWHAZ、ZC3HC1、ZDHHC3、ZMYND11、ZNF205、ZNF385A、ZNF385B、ZNF622、ZSWIM2
Pyroptosis	BAK1、BAX、CASP1、CASP3、CASP4、CASP5、CASP6、CASP8、CASP9、CHMP2A、CHMP2B、CHMP3、CHMP4A、CHMP4B、CHMP4C、CHMP6、CHMP7、CYCS、ELANE、GPX4、GSDMB、GSDMC、GSDMD、GSDME、GZMB、HMGB1、IL18、IL1A、IL1B、IRF1、IRF2、NLRC4、NLRP1、NLRP2、NLRP3、NLRP6、NLRP7、NOD1、PLCG1、PJVK、PRKACA、PYCARD、SCAF11、TINAP、TNF、TP53、TP63、AIM2、GSDMA、IL6、NOD2、TIRAP
Ferroptosis	ABCC1、ACACA、ACO1、ACSF2、ACSL1、ACSL3、ACSL4、ACSL5、ACSL6、AIFM2、AKR1C1、AKR1C2、AKR1C3、ALOX12、ALOX15、ALOX5、ATG5、ATG7、ATP5MC3、BACH1、CARS、CBS、CD44、CHAC1、CISD1、CP、CRYAB、CS、CYBB、DPP4、EMC2、FADS2、FANCD2、FDFT1、FTH1、FTL、FTMT、G6PD、GCLC、GCLM、GLS2、GOT1、GPX4、GSS、HMGCR、HMOX1、HSBP1、HSPB1、IREB2、KEAP1、LPCAT3、MAP1LC3A、MAP1LC3B、MAP1LC3C、MT1G、NCOA4、NFE2L2、NFS1、NOX1、NQO1、NRF2、OTUB1、PCBP1、PCBP2、PEBP1、PGD、PHKG2、PRNP、PROM2、PTGS2、RPL8、SAT1、SAT2、SLC11A2、SLC1A5、SLC39A14、SLC39A8、SLC3A2、SLC40A1、SLC7A11、SQLE、STEAP3、TF、TFRC、TP53、VDAC2、VDAC3、ZEB1
Autophagy	ABL1、ABL2、ACER2、ADRA1A、ADRB2、AKT1、AMBRA1、

	ATF6、ATG101、ATG13、ATG14、ATG2A、ATG2B、ATG5、ATG7、ATM、ATP13A2、ATP6V0A1、ATP6V0A2、ATP6V0B、ATP6V0C、ATP6V0D1、ATP6V0D2、ATP6V0E1、ATP6V0E2、ATP6V1A、ATP6V1B1、ATP6V1B2、ATP6V1C1、ATP6V1C2、ATP6V1D、ATP6V1E1、ATP6V1E2、ATP6V1G1、ATP6V1G2、ATP6V1H、AUP1、BAD、BAG3、BCL2、BCL2L11、BECN1、BMF、BNIP3、BNIP3L、BOK、C9orf72、CALCOCO2、CAMKK2、CAPN1、CAPNS1、CASP1、CASP3、CDC37、CDK5、CDK5R1、CHMP4A、CHMP4B、CISD2、CLEC16A、CLN3、CLU、CPTP、CSNK2A2、CTSA、CTTN、DAP、DAPK1、DAPK2、DAPK3、DAPL1、DCN、DDIT3、DDRGK1、DEPDC5、DEPP1、DHRSX、DNM1L、DRAM1、DRAM2、EEF1A1、EEF1A2、EIF2AK4、EIF4G1、EIF4G2、ELAPOR1、EP300、EPM2A、ERCC4、ERN1、EXOC1、EXOC4、EXOC7、EXOC8、FBXL2、FBXO7、FBXW7、FEZ1、FEZ2、FLCN、FOXK1、FOXK2、FOXO1、FOXO3、FTH1、FTL、FYCO1、FZD5、GAPDH、GATA4、GBA、GFAP、GNAI3、GOLGA2、GPR137、GPR137B、GPSM1、GSK3A、GSK3B、HAX1、HDAC6、HERC1、HGF、HIF1A、HMGB1、HMOX1、HSP90AA1、HSPA8、HSPB1、HSPB8、HTR2B、HTRA2、HTT、HUWE1、IFI16、IFNG、IKBKG、IL10、IL10RA、IL4、IRGM、ITPR1、KAT5、KAT8、KDM4A、KDR、KEAP1、KIF25、KLHL22、KLHL3、LACRT、LAMP1、LAMP2、LAMP3、LAMTOR1、LAMTOR2、LAMTOR3、LAMTOR4、LAMTOR5、LARP1、LEP、LEPR、LGALS8、LRRK2、LRSAM1、LZTS1、MAP1LC3A、MAP1LC3B、MAP1LC3C、MAP3K7、MAPK15、MAPK3、MAPK8、MAPT、MCL1、MEFV、MET、MFN2、MFSD8、MID2、MIR199A1、MIRLET7B、MLST8、MT3、MTCL1、MTDH、MTM1、MTMR3、MTMR4、MTMR8、MTMR9、MTOR、NCOA4、NEDD4、NLRP6、NOD1、NOD2、NPC1、NPRL2、NRBP2、NUPR1、OPTN、ORMDL3、OSBPL7、PAFAH1B2、PARK7、PHB2、PHF23、PIK3C2A、PIK3C3、PIK3CA、PIK3CB、PIK3R2、PIM2、PINK1、PIP4K2A、PIP4K2B、PIP4K2C、PJVK、PLEKHF1、PLK2、PLK3、POLDIP2、PRKAA1、PRKAA2、PRKAB1、PRKAB2、PRKACA、PRKAG1、PRKAG2、PRKAG3、PRKD1、PRKN、PSAP、PTPN22、PYCARD、QSOX1、RAB39B、RAB3GAP1、RAB3GAP2、RAB7A、RAB8A、RALB、RASIP1、RB1CC1、RETREG1、RETREG3、RHEB、RIPK2、RMC1、RNF152、RNF41、RNF5、ROCK1、RPTOR、RRAGA、RRAGB、RRAGC、RRAGD、RUBCN、RUFY4、SCFD1、SCOC、SEC22B、SESN1、SESN2、SESN3、SH3BP4、SH3GLB1、SIRT1、SIRT2、SLC38A9、SMCR8、SMG1、SNCA、SNRNP70、SNX32、SNX5、SNX6、SOGA1、SOGA3、SPTLC1、SPTLC2、SQSTM1、SREBF1、SREBF2、STAT3、STBD1、STING1、STK11、STUB1、SUPT5H、SVIP、SYNPO2、TAB2、TAB3、TBC1D14、TBC1D25、TBK1、

	TEX264、TFEB、TICAM1、TIGAR、TLK2、TMEM150A、TMEM150B、TMEM150C、TMEM39A、TMEM39B、TMEM59、TOMM7、TP53、TP53INP1、TP53INP2、TPCN1、TPCN2、TREM2、TRIB3、TRIM13、TRIM14、TRIM21、TRIM22、TRIM27、TRIM34、TRIM38、TRIM5、TRIM6、TRIM65、TRIM68、TRIM8、TRIML1、TRIML2、TSC1、TSC2、TSPO、UBA5、UBQLN1、UBQLN2、UBQLN4、UCHL1、UFC1、UFL1、UFM1、ULK1、USP10、USP13、USP30、USP33、USP36、UVRAG、VDAC1、VPS13C、VPS13D、VPS26A、VPS26B、VPS35、WAC、WASHC1、WDFY3、WDR24、WDR41、WDR6、WDR81、WIPI2、ZC3H12A、ZKSCAN3、ZMPSTE24
Necroptosis	GLUD1、GLUD2、ALOX15、FTH1、PYG、CAPN1、CASP1、GLNA、BAX、BCL2、FADD、RIPK1、TNF、TNFRSF1A、TRADD、TRAF2、PPIA、CAPN2、HSP90A、IL1A、TNFSF6、TNFRSF6、CASP8、JNK、JAK2、CAMK2、IL1B、IFNG、STAT3、IRF9、TNFSF10、TNFRSF10A、TNFRSF10B、CFLAR、XIAP、BID、AIFM1、TRPM7、IFNAR1、IFNAR2、IFNGR1、IFNGR2、TLR3、TIRP、IFNA、IFNB、TRIF、VDAC1、SLC25A4S、PPID、CYLD、RIPK3、MLKL、TRAF5、TLR4、RBCK1、HMGB1、JAK1、JAK3、TYK2、STAT1、STAT2、STAT4、STAT5A、STAT5B、STAT6、H2A、TNFAIP3、RNF31、CHMP2A、CHMP2B、VPS24、CHMP4A、CHMP4B、CHMP6、VPS4、CHMP1、CHMP5、SMPD1、PYCARD、NLRP3、ZBP1、IL33、FTL、SQSTM1、VDAC2、VDAC3、CHMP7、PGAM5、BIRC2、BIRC3、EIF2AK2、PLA2G4、DNM1L、SPATA2、FAF1、SHARPIN、NOX2、USP21、PARP1、CHMP4C
Cuproptosis	NFE2L2、NLRP3、ATP7B、ATP7A、SLC31A1、FDX1、LIAS、LIPT1、LIPT2、DLD、DLAT、PDHA1、PDHB、MTF1、GLS、CDKN2A、DBT、GCSH、DLST
Parthanatos	PARP、MIF、AIFM1、HSP70、PAAN、ARH3、RNF146、ADPRHL2、OGG1
Entotic cell death	AMPK、ATG5、ATG7、BECN1、CDC42、CDH1、CTNNA1、CYBB、MYH14、PI3KC3、RHOA、RNF146、ROCK、RUBCN、UVRAG
Netotic cell death	ELANE、MMP1、MPO、CAMP、PADI4、EIPA、NCX1、MIA
Lysosome-dependent cell death	ABCA2、ABCB9、ACP2、ACP5、ADGRE2、AGA、AP1B1、AP1G1、AP1M1、AP1M2、AP1S1、AP1S2、AP1S3、AP3B1、AP3B2、AP3D1、AP3M1、AP3M2、AP3S1、AP3S2、AP4B1、AP4E1、AP4M1、AP4S1、ARF1、ARL8B、ARSA、ARSB、ARSG、ASAH1、ATP10B、ATP13A2、ATP6AP1、ATP6V0A1、ATP6V0A2、ATP6V0A4、ATP6V0B、ATP6V0C、ATP6V0D1、ATP6V0D2、ATP6V1H、BLK、BLOC1S1、BLOC1S2、BORCS5、BORCS6、BTK、C12orf4、CBL、CD164、CD300A、CD63、CD68、CD84、CHGA、CLN3、CLN5、CLNK、CLTA、CLTB、

	CLTC、CLTCL1、CLU、CPLX2、CTNS、CTSA、CTSB、CTSC、CTSD、CTSE、CTSF、CTSG、CTSH、CTSK、CTSL、CTSO、CTSS、CTSV、CTSW、CTSZ、DEF8、DNASE2、DNASE2B、ENTPD4、FAM98A、FER、FES、FGR、FLCN、FOXF1、FTH1、FTL、FUCA1、GAA、GAB2、GALC、GALNS、GATA2、GBA、GCC2、GGA1、GGA2、GGA3、GLA、GLB1、GM2A、GNPTAB、GNPTG、GNS、GUSB、HDAC6、HEXA、HEXB、HGS、HGSNAT、HMOX1、HPS6、HSPA8、HYAL1、IDS、IDUA、IGF2R、IL13、IL13RA2、IL4、IL4R、KIF1B、KIT、KXD1、LAMP1、LAMP2、LAMP3、LAMTOR1、LAPTM4A、LAPTM4B、LAPTM5、LAT、LAT2、LGALS9、LGMN、LIPA、LRRK2、LYN、M6PR、MAN2B1、MANBA、MAP1LC3A、MAP6、MCOLN1、MFSD8、MILR1、MRGPRX2、MT3、MYH9、NAGA、NAGLU、NAGPA、NAPSA、NCOA4、NDEL1、NEDD4、NEU1、NPC1、NPC2、NR4A3、PDPK1、PIK3C3、PIK3CD、PIK3CG、PIP4K2A、PIP4K2B、PIP4P1、PLA2G15、PLA2G3、PLEKHM1、PLEKHM2、PPT1、PPT2、PSAP、PSAPL1、PTGDR、PTGDS、RAB34、RAB3A、RAB7A、RAC2、RUBCNL、S100A13、SCARB2、SGSH、SLC11A1、SLC11A2、SLC17A5、SMPD1、SNAP23、SNAPIN、SNX16、SNX4、SORL1、SORT1、SPAG9、SPHK2、SQSTM1、STXBP1、STXBP2、SUMF1、SYK、SYTL4、TCIRG1、TFEB、TMEM106B、TPP1、UNC13D、VAMP7、VAMP8、VPS33A、VPS33B、VPS4A、WASH3P、ZFYVE16
Alkaliptosis	IKBKB、NFKB1、CA9、CHUK、IKBKG、NFKB1A、RELA
Oxeiptosis	PGAM5、KEAP1、AIFM1、NRF2、AIRE
NETosis	MYD88、TLR2、PAD4、PRKCA、PKCB、PRKCZ、NOX3、NOX4、NOX1、CTSG、PRTN3、ELANE、MPO、GSDMD、IL1B、CXCL1、PLA2G7、CXCL8、CDK6、HMGB1、MMP9、AGER、CSF3、TGFB1
Immunogenic_cell_death	ATG5、BAX、CALR、CASP1、CASP8、CD4、CD8A、CD8B、CXCR3、EIF2AK3、ENTPD1、FOXP3、HMGB1、HSP90AA1、IFNA1、IFNB1、IFNG、IFNGR1、IL10、IL17A、IL17RA、IL1B、IL1R1、IL6、LY96、MYD88、NLRP3、NT5E、P2RX7、PDIA3、PIK3CA、PRF1、TLR4、TNF
Anoikis	BRMS1、PTK2、NTRK2、BCL2L11、SRC、CEACAM6、CAV1、AKT1、ITGB1、CEACAM5、EGFR、BCL2、CASP8、SIK1、PTRH2、STAT3、TLE1、DAPK2、CTNNB1、ZNF304、MAPK1、BMF、ITGA5、TP53、MCL1、BCL2L1、CASP3、CDH1、BAD、PIK3CA、PAK1、ITGAV、FN1、MAPK3、PTGS2、BAX、BCAR1、PTEN、ERBB2、ANGPTL4、PDK4、CYCS、BRAF、YAP1、ANKRD13C、ITGA2、ANXA5、BIRC5、MTOR、TIMP1、BDNF、CSPG4、BSG、AKT2、STK11、IGF1、IGF1R、ITGA6、ILK、CFLAR、RHOA、HIF1A、DAP3、MYBBP1A、TLE5、ITGA3、PTK2B、CCND1、CTTN、CALR、ATF4、CDCP1、PLAUR、

	SKP2、CHEK2、HGF、E2F1、EGF、PIK3CG、ITGB4、DAPK1、MAPK8、PIK3R1、PIK3R3、MAP2K1、CXCL12、LGALS3、FBXW7-AS1、BAK1、ABHD4、CD44、ITGA4、FADD、PHLDA2、TGFB1、HMCN1、MMP2、CEBPB、CEMIP、CDKN3、CBL、CASP9、SFN、MTDH、PRKCA、TNFRSF10B、CXCL8、MIR200C、AR、CDKN2A、CPT1A、PIK3CB、CLDN1、MIR204、MIR26A1、CDKN1A、CDKN1B、KLF12、NTRK1、PLAU、MYC、SMAD4、PLK1、MUC1、LGALS1、PYCARD、SESN2、ITGB3、KRAS、THBS1、BID、HRAS、CDK11B、CDK11A、XIAP、PPARG、IL6、MIR145、CCR7、MSLN、RAC1、GRHL2、BIRC3、NOTCH1、RHOG、CCAR2、NQO1、MMP13、FAS、MTA1、MYO5A、EDA2R、CCN6、MMP9、ABL1、MAPK11、SOD2、PTHLH、PDGFB、GLI2、EZH2、RIPK1、CXCR4、HMGA1、SIK2、TNFSF10、ANGPTL2、S100A4、NTF3、ETV4、MIR21、MIR124-1、HTRA1、LATS1、CEACAM3、EIF2AK3、LAMC2、LAMA3、LAMB3、CDH2、CSNK2A1、EDIL3、ZEB2、TLN1、EPHA2、SIRT3、OLFM3、CLU、SPINK1、CPEB2、NAT1、TSG101、MIR200A、MIR6744、SERPINA1、AKT3、RELA、TNFRSF1A、FASLG、AFP、ITGA8、NOX4、PBK、SATB1、CD63、EEF1A1、LTB4R2、MAVS、HRC、CCN2、RHOB、PPP1R13B、PLG、MET、RAF1、PARP1、PRKCQ、BRCA2、RB1、SP1、HAVCR2、DOCK1、VTN、INHBB、PDCD4、PRPF4B、RANBP9、SESN1、SESN3、CD24、ZBTB7A、MIR141、ELANE、KDR、MDM2、NFE2L2、ZEB1、KL、PRKCI、CRYAB、EPHB6、FGF2、HK2、LTF、IQGAP1、MGAT5、SDCBP、ABHD2、SPIB、TRIM31、MIR1827、PDGFRB、PLAT、TLR3、NRAS、ROCK1、PAK4、VEGFA、CASP10、PIN1、IL1RAP、UBE2C、YWHAZ、TWIST1、BMP6、BNIP3L、ELK1、KDM3A、PRDX4、BNIP3、LMO3、ZNF32、MIR200B、MIR525、MIR363、TUBB3、HSP90B1、SLC2A1、HMOX1、PTPN11、PRKACA、PAK3、CD36、PIK3R2、PPP2CA、CASP6、CDH3、EEF2K、LRP1、PAK2、PTK6、LPAR1、TCF7L2、CEACAM1、GDF2、GLO1、IL17A、RBL2、SIRPA、TRAF2、ADCY10、VPS37A、TNFRSF12A、APOBEC3G、BAG1、COL13A1、MNX1、RAD9A、IFI27、MEGF11、ITPRIP、BCL2L15、SNAI2、PTPN1、NOTCH3、GLUD1、SIRT1、FASN、MYH9、RPS6KB1、TPM1、PPP2R1A、COL4A2、CTNND1、CD151、MMP11、ARHGEF7、PPP2R2A、SEMA7A、PPP2R5A、BST2、CCN1、PPP2R2D、CCDC178、MIR10A、MIR30B、MIR30C1、SHC1
Paraptosis	CAMK2B、PRKACG、MARK4、SSTR5、TAAR5、USP10、PRKAG3、HACD2、NT5C、INSRR、SSTR3、TAAR9、HSPB8、PLPP2、G6PC2、GUCY2EP、CDK4、RGR、ADGRG1、UQCRC1、TNK2、RNF181、MKNK2、UBE2U、MYLK、CTDSP2、LCK、GPR15、ATP23、LPAR1、PI4KB、DSTYK、CFD、PPP3CA、

	CCR4、PRAG1、CDKN3、GPR153、DDIT3、MAPK8、MAP2K2、MAPK1、MAPK14、IGF1R、PDCD6IP、CASP9、ERN1、ATF6、XBP1、AKT1、EIF2S1、HSPA5、CASP4、CASP3、CASP7、ITPR3、RYR1、RYR2、MCU、TNFRSF19、PDCD5、CSF1、TP53、NFKB1、PEBP1、PHB
Methuosis	CSNK2A1、RAC1、ARF6、GIT1、MTOR、MET、PFKFB3、MIR199A1
Entosis	AR、TP53、ROCK1、PCK2、TNFSF10、MTUS2、AURKA、KIF2C、RHOA、MTOR、EZR、GZMB、MRTFA、PTK2、DIAPH1、LPAR2、MAP1LC3A、PIKFYVE、CTTN、MCOLN1、FOXO1、CTNNA1、CXCL8
